# The Mechanism of NEDD8 Activation of CUL5 Ubiquitin E3 Ligases

**DOI:** 10.1074/mcp.RA120.002414

**Published:** 2021-01-06

**Authors:** Ryan J. Lumpkin, Alla S. Ahmad, Rachel Blake, Christopher J. Condon, Elizabeth A. Komives

**Affiliations:** Department of Chemistry and Biochemistry, University of California, San Diego, La Jolla, California, USA

**Keywords:** hydrogen-deuterium exchange mass spectrometry, ubiquitin ligase, protein complex, post-translational modifications, ASB9, ankyrin repeat and SOCS box protein 9, CKB, creatine kinase brain-type, CRLs, Cullin RING E3 ligases, ELOB/C, elongin B/elongin C, HDX-MS, hydrogen–deuterium exchange mass spectrometry, LC MS/MS, nanospray liquid chromatography coupled to tandem mass spectrometry, RBRLs, RING between RING E3 ligases, SOCS, suppressor of cytokine signaling, Ub, ubiquitin

## Abstract

Cullin RING E3 ligases (CRLs) ubiquitylate hundreds of important cellular substrates. Here we have assembled and purified the Ankyrin repeat and SOCS Box protein 9 CUL5 RBX2 ligase (ASB9-CRL) *in vitro* and show how it ubiquitylates one of its substrates, CKB. CRLs occasionally collaborate with RING between RING E3 ligases (RBRLs), and indeed, mass spectrometry analysis showed that CKB is specifically ubiquitylated by the ASB9-CRL-ARIH2-UBE2L3 complex. Addition of other E2s such as UBE2R1 or UBE2D2 contributes to polyubiquitylation but does not alter the sites of CKB ubiquitylation. Hydrogen–deuterium exchange mass spectrometry (HDX-MS) analysis revealed that CUL5 neddylation allosterically exposes its ARIH2 binding site, promoting high-affinity binding, and it also sequesters the NEDD8 E2 (UBE2F) binding site on RBX2. Once bound, ARIH2 helices near the Ariadne domain active site are exposed, presumably relieving its autoinhibition. These results allow us to propose a model of how neddylation activates ASB-CRLs to ubiquitylate their substrates.

## Highlights

• ARIH2 is required for ASB9CRL to polyubiquitylate 4/18 lysines on one creatine kinase subunit.
• HDX-MS reveals long-range allosteric opening of a cleft in CUL5 where the ARIH2 RBRL binds.
• HDX-MS reveals that neddylation of CUL5 alters the RBX2 conformation away from binding the E2∼NEDD8.
• HDX-MS reveals opening of the ARIH2 active site upon binding CUL5, thus releasing its autoinhibition.

## Graphical Abstract

**Figure:**
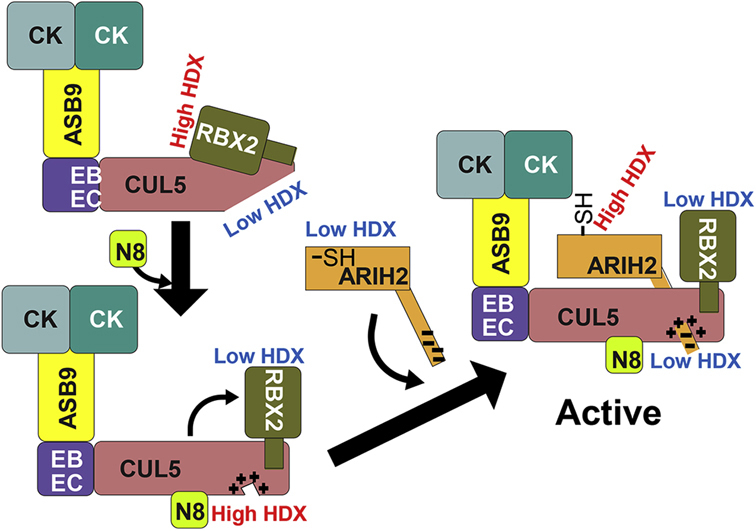

## In Brief

Hydrogen-deuterium exchange mass spectrometry (HDX-MS) reveals how a CUL5 E3 ligase is activated for polyubiquitylation of its substrate.

**Fig. 1:**
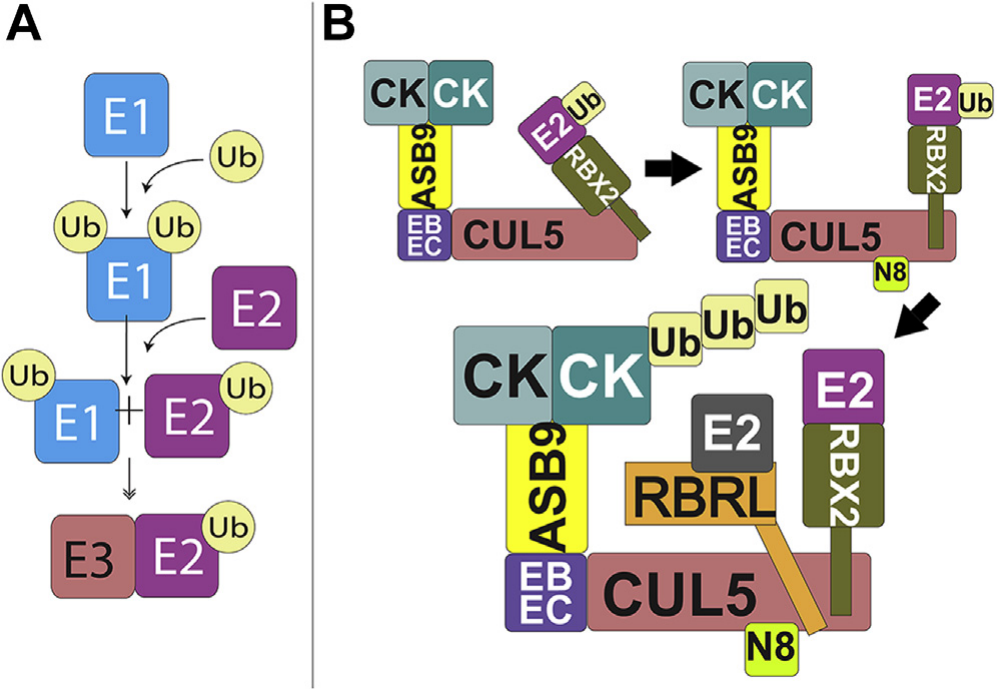
**Schematics of the ubiquitylation cascade as mediated by the ASB9 CUL5 E3 ligase.***A*, ubiquitin is activated through a three-step enzymatic cascade. The ubiquitin-activating enzyme (E1) binds ATP and catalyzes adenylation of ubiquitin. The active-site cysteine on E1 attacks the Ub-AMP complex to form a thioester bond. Through a *trans*-thioesterification reaction, ubiquitin is transferred to the active-site cysteine on a ubiquitin-conjugating enzyme (E2). Ubiquitin ligases (E3) facilitate the highly specific covalent attachment of activated ubiquitin (Ub) to bound substrate proteins through an isopeptide bond on an exposed lysine residue. *B*, schematic showing the states of the ASB9-CRL explored in this work: unactivated, activated by NEDD8, and activated by NEDD8 with ARIH2 present. The protein abbreviations and colors are consistent throughout the manuscript: creatine kinase brain-type (CKB, *two-tone teal*); ankyrin and SOCS-box protein 9 (ASB9, *yellow*); elongins B and C (ELOB/C, *purple*); cullin 5 (CUL5, *salmon*), ring box protein 2 (RBX2, *olive*); the ubiquitin conjugating enzyme bound to RBX2 (UBE2D1/2, *magenta*); ubiquitin (Ub, *tan*); Ariadne RBR E3 ubiquitin protein ligase 2 Ring between Ring ligase (ARIH2 RBRL, *orange*); the ARIH2 RBRL ubiquitin conjugating enzyme (UBE2L3, *gray*). The Ub is transferred from UBE2L3 to ARIH2 prior to transfer to the substrate.

Ubiquitin (Ub) is a posttranslational modification that is installed by an activating enzyme (E1), which transfers the Ub to a conjugating enzyme (E2), and a ligase (E3), which brings the target substrate together with the E2 (Fig. 1*A*). Association of substrates with E3s leads to the covalent attachment of Ub onto substrate lysines and poly-Ub chains. Formation of poly-Ub chains may involve more than one E2 enzyme (1). Both mono-Ub and the various poly-Ub chains lead to distinct signals. Ubiquitin monomers can be polymerized at any of seven lysine residues or the first methionine residue. The manner in which differential ubiquitin signaling is achieved by the differential interactions of distinct chain linkages with the proteasome, ubiquitin-binding proteins and deubiquitinases (DUBs) is referred to as the ubiquitin code (1, 2). The K48 and K63 linkages are most abundant, and these are found as pure chains, mixed chains, and branched chains in mammalian cells (3). Typically, proteasome targeting is thought to be the function of K48-linked chains, although others such as K11- or K29-linked chains as well as multiple short chains can also function as proteasomal signals (4, 5).

E3 ligases fall into three major groups: homologous to E6-AP carboxyl terminus (HECT), really interesting new gene (RING), and ring-between-RING ligases (RBRL). RING E3s account for approximately 90% of known Ub ligases, and Cullin-RING ligases (CRLs) are the most common RING E3s. CRLs are multisubunit complexes consisting of a substrate receptor, adaptor proteins, a Cullin protein (CUL1-5, CUL7), and a RING protein (RBX1 or RBX2). The RING protein associates with Ub-bound E2 enzymes to facilitate the direct transfer of Ub from the E2 to bound substrate proteins. CUL5 interacts specifically with RBX2 (6).

CUL2 and CUL5 have been shown to bind elongin B and elongin C (ELOB/C), through which CUL2 binds vHL-box substrate receptors and CUL5 binds suppressor of cytokine signaling (SOCS)-box substrate receptors (7). Here we focus on the 18-member ankyrin repeat and SOCS-box (ASB) adapter family; a large class of E3 ligase substrate receptors (6). Each of the 18 ASB family members binds approximately ten proteins (8), which are possible substrates for ubiquitylation, although that remains to be demonstrated. The ASB proteins contain an ankyrin repeat domain (9, 10), presumably involved in substrate binding, and a SOCS-box domain, which contains the conserved BC-box and CUL5-box motifs for binding to ELOB/C. ASB9 was shown to bind creatine kinases (CKB) and to induce their ubiquitylation and degradation in a SOCS-box-dependent manner (11, 12). ASB9 binds a dimer of CKB at the first ankyrin repeat (13). Structure determination of the ASB9-ELOB/C complex (14) and modeling of CKB, CUL5, and RBX1 led to models of the full ligase that depicted CKB bound to ASB9 approximately 70 Å away from the E2∼Ub complex on RBX1 (15). We recently reported on a part of the structure of this ligase (16). A structure has recently been solved for neddylated CUL1-SKP1, β-TRCP, and a substrate peptide showing by strategic cross-linking how the RBX1-bound ubiquitylated E2D reaches the substrate (17). This structure reveals one of the many possible functions of neddylation wherein the NEDD8 forms an interaction with the E2D.

Some CRLs have been shown to have two modes of action, one in which the E2 attached to the RBX is responsible for Ub transfer and a second in which an RBRL, either ARIH1 or ARIH2, performs the Ub transfer. The ARIHs are RBRLs with characteristics of both HECT and RING ligases (18). These E3s contain two RING domains and an in-between-RING domain, but unlike RING ligases, the E3s associate with a cysteine-reactive E2 enzyme, UBE2L3, which transfers Ub to the catalytic cysteine on the RBRL (19). ARIH1 has been shown to interact with CUL1-4 and is important for Ub transfer to some CRL substrates (20). ARIH2 specifically interacts with a basic patch on CUL5 *via* an acidic N terminus of ARIH2 (21) and has been shown to be essential for Ub transfer by a CUL5 ligase to the substrate APOBEC3G (22). Binding to CRLs is thought to relieve autoinhibition of the RBRLs (21), enabling the RBRL to ubiquitinate the substrate on the CRL (20). ARIH2 was shown to coprecipitate with ASB9 in pull-down proteomics studies (23) and recently was shown to help ubiquitinate CKB in the presence of the ASB9-CRL (22). Structures of ARIH2 bound to CUL5 are not yet available, and the mechanism by which neddylation of CUL5 promotes binding of ARIH2 remains unknown.

Here, we present biochemical, structural, and biophysical data on the purified ASB9-CRL we have assembled *in vitro*. We show that the ASB9-CRL does not significantly ubiquitylate CKB in the absence of ARIH2-UBE2L3∼Ub. The requirement of ARIH2 for efficient ubiquitylation of CKB distinguishes the mechanism of the CUL5-containing ASB9-CRL from the CUL1-containing ligase for which a structure was recently determined (17). Neddylation of CUL5 is required for high-affinity binding of ARIH2, consistent with hydrogen–deuterium exchange mass spectrometry (HDX-MS) experiments showing that neddylation allosterically exposes the ARIH2 binding site on CUL5. Neddylation also reduces solvent exposure of the NEDD8 E2, UBE2F binding site on RBX2, consistent with the need to exchange UBE2F with another E2 for ubiquitylation once neddylation is completed. When added to the CKB-bound ASB9-CRL, ARIH2-UBE2L3∼Ub is sufficient to add multiple Ub moieties to four lysines on CKB, and addition of other E2s such as UBE2R1 or UBE2D2 modulates the polyubiquitin linkage activity of the ligase, but not the sites of ubiquitylation on CKB. HDX-MS experiments show that binding of ARIH2 to CUL5 allosterically triggers opening of its autoinhibited Ariadne domain. These results provide a mechanism of how neddylation of the ASB9-CRL promotes ARIH2 binding and RBX2 E2 selectivity as well as how the ARIH2 is activated upon binding to CUL5.

## Experimental Procedures

### Expression Vectors

Human ASB9-1 in pNIC-CTHF was obtained from the Structural Genomics Consortium and subcloned into pHis8 (Kan^R^) with an N-terminal 8xHis tag. Human CKB was subcloned into pET11a (Amp^R^) as previously described (13). Human ELOB (full-length) and ELOC (17–112) were obtained in pACYC (Cam^R^) (Structural Genomics Consortium). Human CUL5 and mouse RBX2 were obtained in pRSFDuet (Kan^R^) with an N-terminal His tag, TEV cleavage site, and GB1 tag on CUL5 (gift from Nevan Krogan). 6xHis-GB1-TEV-CUL5 was subcloned into pET28a (Kan^R^), and RBX2 was subcloned into pET11a (Amp^R^).

Human UBA1 in pET21b (Amp^R^) with an N-terminal 6xHis tag was obtained from Addgene (Plasmid #34965). Human UBE2D2 in pET-SUMO (Amp^R^) with an N-terminal 6xHis tag and SUMO solubility tag was obtained from Addgene (Plasmid #60443) and was subcloned into pET28a with an N-terminal 6xHis tag and TEV cleavage site. Human UBE2R1 in pDEST17 with an N-terminal 6xHis tag was obtained from Addgene (Plasmid #18674). Human Ub in pcDNA3 (Amp^R^) with an N-terminal HA tag was obtained from Addgene (Plasmid #18712) and subcloned into pET28a (Kan^R^) with an N-terminal 6xHis tag and TEV cleavage site. Human NAE1 and UBA3 were obtained in pGEX4T1 (Amp^R^) with a GST tag (gift from Brenda Schulman). Human UBE2F in pDEST17 (Amp^R^) with an N-terminal 6xHis tag was obtained from Addgene (Plasmid #15800) and was subcloned into pET28a (Kan^R^) with an N-terminal 6xHis tag and TEV cleavage site. Human NEDD8 in pcDNA3 (Amp^R^) with an N-terminal HA tag was obtained from Addgene (Plasmid #18711) and was subcloned into pET28a (Kan^R^) with an N-terminal His tag. Codon-optimized human ARIH2 was synthesized with an N-terminal 6xHis tag, MBP solubility tag, and TEV cleavage site, and it was subcloned into pET11a (Amp^R^) (GENEWIZ, Inc). Codon-optimized human UBE2L3 was synthesized and subcloned into pET28a (Kan^R^) with an N-terminal 6xHis tag and TEV cleavage site. TEV Protease was obtained in pRK793 (Amp^R^) with an N-terminal MBP solubility tag, TEV cleavage site, and 6xHis tag (gift from César Ramirez-Sarmiento).

### Protein Expression

ASB9 was coexpressed with ELOB/C and/or CKB using sequential transformation with appropriate vectors (above) into BL21 *Escherichia coli* cells for ASB9-CKB, ASB9-ELOB/C, and ASB9-CKB-ELOB/C protein complexes. Vectors containing ELOB/C, CKB, or both were transformed into competent BL21 cells after which those cells were made competent again. ELOB/C in pACYC was selected for by chloramphenicol (CAM) resistance, and CKB in pET11a was selected for by ampicillin (AMP) resistance. The vector for expression of ASB9 was transformed into ELOB/C + CKB-containing BL21 cells and plated on a kanamycin (KAN)-CAM-AMP LB agar plate. The pET28a vector for expression of CUL5 and the pET11a vector for expression of RBX2 were cotransformed into ELOB/C-containing BL21 cells and plated on a KAN-CAM-AMP LB agar plate for coexpression of CUL5/RBX2/ELOB/C. Vectors for expression of UBE1 and Ub were cotransformed into BL21 cells and plated on a KAN-AMP LB agar plate for coexpression of UBE1/Ub. Vectors for expression of UBE2D2, UBE2L3, UBE2F, Ub, and Nedd8 were transformed individually into BL21 cells and plated on KAN LB agar plates. Vectors for expression of NAE1/UBA3, UBE2R1, and ARIH2 were transformed individually into BL21 cells and plated on AMP LB agar plates.

All proteins were expressed as follows. A 5 ml M9-ZN (1.5 × M9 salts, NZ-Amine media, 0.8% dextrose, 1 mM MgSO_4_, 0.2 mM CaCl_2_) overnight culture was inoculated with a single colony from the plate. A 20 ml M9-ZN starter culture was inoculated with 2 ml of the overnight culture and grown for 3 h at 37 °C. The 1 L M9-ZN growth culture was inoculated with the entire 20 ml starter culture and grown until OD_600_ = 0.8. After placing the cultures on ice for 15 min, protein expression was induced by addition of IPTG to a final concentration of 0.5 mM, and the cultures were transferred to an 18 °C incubator for 16 to 18 h. Because RBX2 and ARIH2 contain Zn binding domains, the cultures containing either of those proteins were brought to 200 μM Zn by addition of a 1 M solution of ZnCl_2_ just prior to induction.

### Protein Purification

Cells from 1 L of culture were pelleted by centrifugation at 3500 xg for 10 min, then resuspended in 40 ml resuspension buffer (50 mM Tris-HCL pH 8.0, 100 mM NaCl, 10 mM imidazole pH 8.0, 2 mM β-mercaptoethanol, 5% glycerol) with protease inhibitor cocktail (Sigma P2714) and 5 mM PMSF. Cells were lysed on ice by sonication with ten 30 s pulses with 45 s cooldown between each pulse. The lysate was clarified by centrifugation at 20,000 xg for 45 min. The clarified lysate was incubated with 2 ml Ni-NTA (in resuspension buffer) for 2 h at 4 °C with rocking. Ni-NTA beads were pelleted by centrifugation at 700*g* for 5 min. The supernatant was discarded, and the beads were washed with 10 ml wash buffer (50 mM Tris-HCL pH 8.0, 100 mM NaCl, 25 mM imidazole pH 8.0, 2 mM β-mercaptoethanol, 5% glycerol) for 30 min at 4 °C. Ni-NTA beads were again pelleted by centrifugation at 700*g* for 5 min. The supernatant was discarded, and the beads were washed with 10 ml elution buffer (50 mM Tris-HCL pH 8.0, 100 mM NaCl, 250 mM imidazole pH 8.0, 2 mM β-mercaptoethanol, 5% glycerol) for 30 min at 4 °C. Ni-NTA beads were again pelleted by centrifugation at 700*g* for 5 min. The supernatant was transferred to a 12 to 14 kDa dialysis bag and dialyzed overnight in dialysis buffer (20 mM Tris-HCL pH 8.0, 100 mM NaCl, 5% glycerol, 1 mM DTT). Samples were concentrated to 2 ml and purified using size-exclusion chromatography (SEC) over a Superdex S200 16 × 600 column in dialysis buffer. Peak fractions were combined and concentrated to 5 μM for analysis by HDX-MS.

Neddylation reactions following purification of all necessary components resulted in only partial neddylation of CUL5-containing complex. We overcame this limitation by neddylating the CUL5-containing complex during its purification. Lysates for NAE1/UBA3 and NEDD8 were mixed with 10 mM Mg-ATP to form the NAE1/UBA3∼NEDD8 adduct, which was then purified by Ni-NTA chromatography taking advantage of the His-tag on NEDD8 and dialyzed. The lysate containing the CUL5 complex, the lysate containing UBE2F (the E2 for CUL5 neddylation), and the NAE1/UBA3∼NEDD8 were then mixed with 10 mM ATP at 4 °C. Full neddylation of CUL5 occurred within 30 min at 4 °C. The neddylated CUL5 complex was purified from NAE1/UBA3, UBE2F, and excess NEDD8 by SEC on Superdex S200 in dialysis buffer.

ARIH2 and CUL5 were evaluated for binding through SEC. ARIH2 was mixed with an equimolar amount of either ELOB/C-CUL5(NEDD8)-RBX2 or ELOB/C-CUL5-RBX2, and then the resulting mixture was separated by size exclusion. While the sample with neddylated CUL5 eluted as a mostly homogenous peak, the nonneddylated sample eluted in two clearly separate peaks. SDS-PAGE of the SEC fractions indicated that ARIH2 coeluted preferentially with neddylated CUL5.

GB1 was cleaved from CUL5, and MBP was cleaved from ARIH2 by adding TEV to the proteins at a 1:10 M ratio and incubating at 4 °C for 24 h. TEV and the cleaved tags were removed by size exclusion over a Superdex S200 Increase 10/300 column in 20 mM Tris-HCl pH 8.0, 100 mM NaCl, 5% glycerol, 1 mM DTT.

Sample compositions were identified and characterized according to the presence of the desired proteins as assessed by SEC, SDS-PAGE (10–15% acrylamide gels), and nanospray LC MS/MS on a Lumos mass spectrometer after trypsin digestion.

ELOB/C-CUL5-RBX2, ELOB/C-CUL5(NEDD8)-RBX2, ARIH2, and ELOB/C-CUL5(NEDD8)-RBX2-ARIH2 protein samples were individually prepared for analysis by HDX-MS. ELOB/C-CUL5-RBX2, ELOB/C-CUL5(NEDD8)-RBX2, and ARIH2 were expressed and purified as described above. ELOB/C-CUL5(NEDD8)-RBX2-ARIH2 was formed by combining the ELOB/C-CUL5-RBX2 and ARIH2 following neddylation of CUL5. The combined complex was isolated by size exclusion over a Superdex S200 Increase 10/300 column in 20 mM Tris-HCl pH 8.0, 100 mM NaCl, 5% glycerol, 1 mM DTT.

### Hydrogen–Deuterium Exchange

HDX-MS experiments were conducted using a Waters Synapt G2Si mass spectrometer equipped with a nanoACQUITY ultrahigh-pressure liquid chromatography (UPLC) system equipped with H/DX technology and a LEAP H/D-X PAL liquid handling system as previously described (24). The H_2_O buffer was composed of 20 mM Tris-HCl, pH 8.0, 100 mM NaCl, 5% glycerol, and 1 mM DTT, matching the size-exclusion buffer used in the final stage of purification for each protein sample. This buffer was lyophilized and resuspended in D_2_O (D_2_O Buffer). A 4 μl portion of a 5 μM protein sample was incubated for 5 min at 25 °C and then mixed with 56 μl of H_2_O Buffer as a control or D_2_O buffer for deuteration times of 15 s, 30 s, 45 s, 60 s, or 120 s. Measurement of amide exchange during this time regime best captures allosteric transitions (25).The reaction was quenched with 60 μl of quench buffer (3 M guanidine, 0.1% formic acid, pH 2.66) at 0 °C. A portion of the quenched sample (50 μl) was injected into a sample loop and subsequently digested on an in-line pepsin column (Immobilized Pepsin, Pierce Inc) at 15 °C. The peptides were captured on a BEH C18 Vanguard precolumn and separated by analytical chromatography (Acquity UPLC BEH C18, 1.7 μM, 1.0 × 50 mm, Waters Corporation) using a 7 to 85% acetonitrile in 0.1% formic acid over 7.5 min and electrosprayed into the Waters SYNAPT G2Si quadrupole time-of-flight mass spectrometer. The mass spectrometer was set to collect data in the Mobility, ESI+ mode; mass acquisition range of 200 to 2000 (m/z); scan time 0.4 s. Continuous lock mass correction was accomplished with infusion of leu-enkephalin (m/z = 556.277) every 30 s (mass accuracy of 1 ppm for calibration standard). For peptide identification, the mass spectrometer was set to collect data in MS^E^, ESI+ mode instead.

### Experimental Design and Statistical Rationale

The peptides were identified from triplicate MS^E^ analyses of 5 μM ELOB/C-CUL5(NEDD8)-RBX2 and ARIH2 samples using PLGS 2.5 (Waters Corporation). Peptide masses were identified using a minimum number of 250 ion counts for low-energy peptides and 50 ion counts for their fragment ions. Peptides masses were identified using a minimum number of 250 ion counts for low-energy peptides and 50 ion counts for their fragment ions.

The peptides identified in PLGS were then used to analyze the deuterium uptake in DynamX 3.0 (Waters Corporation) by first applying additional filters in DynamX 3.0 including a cutoff score of 6.5, minimum products per amino acid of 0.2, maximum MH+ error tolerance of 5 ppm, retention time standard deviation of 5%, and requiring that the peptide be present in at least two of the three peptide identification runs. Every mass envelope was manually checked. The deuterium uptake for each peptide was calculated by comparing the centroids of the mass envelopes of the deuterated samples *versus* the undeuterated controls (26). For all HDX-MS data, at least two biological replicates were analyzed, each with three technical replicates. Data are represented as mean values ± SEM of three technical replicates due to processing software limitations; however, biological replicates were highly reproducible due to use of the LEAP robot for all experiments. The deuterium uptake was corrected for back exchange using a global back-exchange correction factor (typically 25%) determined from the average percent exchange measured in disordered termini of the sample proteins (27). ANOVA analyses and *t*-tests with a *p*-value cutoff of 0.05 implemented in the program, DECA (github.com/komiveslab/DECA), were used to determine the significance of differences between HDX data points (28). The peptides reported on the coverage maps are actually those from which deuterium uptake data were obtained. Deuterium uptake plots were plotted in DECA as number of deuterons incorporated *versus* time (min). The Y-axis limit for each plot reflects the total possible number of amides within the peptide that can exchange. The uptake curves were fitted with an exponential curve for ease of viewing.

### Ubiquitylation Reactions

Ubiquitylation reactions were carried out in a buffer containing 20 mM Tris-HCl pH 7.5, 5 mM MgCl_2_, 0.5 mM DTT, and 2 mM ATP with 0.5 μM UBE1, 5 μM UBE2D2 or UBE2R1 and/or ARIH2+UBE2L3, 100 to 200 μM Ub, and 5 μM ASB9-CRL as previously described (29). Reactions were incubated at 37 °C for 3 h to ensure complete reaction (12), although substantial ubiquitin transfer was observed by 20 min. Reactions were subsequently quenched with reducing SDS-PAGE buffer, boiled at 90 °C for 10 min prior to SDS PAGE.

Thioester transfer of ubiquitin between UBE1 and UBE2D2 or UBE2R1 and/or ARIH2+UBE3L3, as well as the covalent ubiquitylation of CKB, was measured by monitoring the molecular weights of the proteins as previously described (30). Samples were separated by 10% polyacrylamide SDS-PAGE in replicate for parallel detection by Coomassie Blue staining and by anti-Ub western blotting using standard approaches. The blot was incubated overnight with rocking at 4 °C with mouse anti-Ub Antibody (Sigma 042691GS) diluted 1:4500 in blocking buffer, rabbit anti-NEDD8 antibody (CST 2754) diluted 1:4500 in blocking buffer, or rabbit anti-creatine kinase B type antibody (Abcam ab38211) diluted 1:500 in blocking buffer. The blot was washed three times with TTBS (TBS with 0.05% Tween) and then incubated with secondary antibody anti-mouse IgG (H + L), HRP conjugate (Promega W4021) or anti-rabbit IgG (H + L), HRP conjugate (Promega W4011) for 1 h with rocking at 23 °C. The blot was washed twice with TTBS and finally with TBS, developed by incubation with Bio-Rad Clarity Western ECL Substrate (1705061) for 5 min at 23 °C and imaged using a Bio-Rad Chemi-Doc. The colorimetric blot default protocol was used to image the molecular weight ladder and the chemi high-resolution blot default protocol was used to detect chemiluminescence with exposures from 1 to 10 s.

### ImageJ Gel Analysis

Coomassie stained gels and anti-CKB blots were imported into ImageJ (31). The background signal was subtracted from each lane, areas were calculated from each peak, and data were plotted gel mobility *versus* intensity.

### NanoLC Mass Spectrometry for Ubiquitin Analysis

Ubiquitylation reaction samples were electrophoresed on 10% Tris-SDS poly-acrylamide gels in SDS gel running buffer for 5 min at 180 V. The gel lanes were excised from the bottom of the stacking gel to the dye front. For mass spectrometry of individual gel bands, samples were separated on 4 to 15% Mini-PROTEAN TGX Precast Protein Gels (Bio-Rad 4561083) in SDS gel running buffer for 60 min at 180 V. After staining for 10 min in 0.25% w/v Coomassie Blue/40% MeOH/10% acetic acid and destaining for 10 min in 20% MeOH/10% acetic acid, the gel bands were excised, cut into small pieces, and washed according to standard procedures (32). The proteins were acetylated, then the cysteines were reduced and alkylated with iodacetamide, and finally the proteins were digested with trypsin for 45 min at 4 °C, then 37 °C overnight. The supernatant containing the tryptic peptides was collected, and remaining peptides were extracted from the gel according to standard procedures (32). The samples were dried in a speed vac and stored at −20 °C until analysis.

Trypsin-digested samples were analyzed by UPLC coupled with tandem mass spectroscopy (LC-MS/MS) using nano-spray ionization on an Orbitrap fusion Lumos hybrid mass spectrometer (Thermo) interfaced with nano-scale reversed-phase UPLC (Thermo Dionex UltiMate 3000 RSLC nano System) using a 25 cm, 75-micron ID glass capillary packed with 1.7-μm C18 (130) BEHTM beads (Waters corporation) and separated on a linear gradient (5–80%) of ACN at a flow rate of 375 nl/min for 1 h in 0.1% formic acid. Mass spectrometer parameters are as follows; an MS1 survey scan using the orbitrap detector (mass range (m/z): 400 to 1500 (using quadrupole isolation), 120,000 resolution setting, spray voltage of 2200 V, ion transfer tube temperature of 275 C, AGC target of 400,000, and maximum injection time of 50 ms) was followed by data-dependent scans (top speed for most intense ions, with charge state set to only include +2 to 5 ions, and 5 s exclusion time, while selecting ions with minimal intensities of 50,000 at which the collision event was carried out in the high-energy collision cell (HCD collision energy of 30%), and the fragment masses were analyzed in the ion trap mass analyzer (with ion trap scan rate of turbo, first mass m/z was 100, AGC Target 5000, and maximum injection time of 35 ms). Protein identification and label-free quantification were carried out using Peaks Studio 8.5 (Bioinformatics solutions Inc) The Human Uniprot Database (May 10, 2020) was searched with trypsin unspecific protease specificity allowing three missed cleavages. The fixed modifications were carbamidomethylation (C), acetylation (K, N-term), and the variable modifications were ubiquitin (K) and PEAKS PTM open search. The precursor mass tolerance was 40 ppm and the fragment mass tolerance was 0.5 Da. The score cutoff was −10 log P > 15. Digestion of a ubiquitinylated peptide by trypsin leaves a “digly” modification with a mass of 114.04 Da on the ubiquitylated lysine (33). To quantify the amount of Ub at each lysine in CKB, the sum of the areas of all peptides with a digly at a particular lysine was divided by the sum of the areas for all peptides covering that lysine, and these values were used for relative comparison between samples.

### Homology Modeling

A model of ASB9-CRL with neddylated CUL5 was prepared by superimposing homologous domains of known structures to position each component in PyMOL. The CKB-ASB9-ELOB/C-CUL5NTD structure was described in Lumpkin *et al.*, 2020 (16). To orient the CUL5CTD relative to CUL5NTD, CUL2 from CUL2-RBX1-ELOB/C-VHL (5N4W) was first aligned to residues 306 to 387 on CUL5NTD in the starting model using PyMOL. CUL5CTD from CUL5CTD(NEDD8)-RBX1 (3DQV) was aligned to residues 383 to 424 on CUL2 from CUL2-RBX1-ELOB/C-VHL (5N4W). Following orientation of the two halves of CUL5, CUL2-RBX1-ELOB/C-VHL was removed from the model. Residues 208 to 238 of RNF4 in RNF4-UBE2D1-Ub (4AP4) were aligned to RBX1 to position UBE2D1-Ub, and RNF4 was removed. Prior to model refinement, sequences extracted from the PDBs were aligned to the full-length human sequences for each protein using the pairwise alignment tool EMBOSS Water from EMBL-EBI. The model by MODELLER (32) is used with the sequence alignments to minimize loop energies, fill sequence gaps, correct solubility mutations, and to model RBX2 from RBX1. MODELLER 9.23 was run using the default automodel class. The model with the lowest MOLPDF score of the ten models generated was selected as the final model.

ARIH2 was aligned to the sequence of ARIH1 extracted from the crystal structure of the ARIH1-UBE2L3-Ub complex (PDB 5UDH) using the pairwise alignment tool EMBOSS Water from EMBL-EBI. Zinc-chelating residues C186, C189, C203, H205, C208, C211, C231, C236, C276, C281, C297, C299, C304, C307, H312, C317, C344, C347, C357, C362, C367, C372, C375, H382, and C389 in ARIH1 correspond to residues C139, C142, C156, H158, C161, C164, C183, C188, C228, C233, C249, C252, C257, C260, H265, C270, C297, C300, C310, C315, C318, C323, C326, H333, and C340 in ARIH2. The two protein sequences were aligned so that the zinc-chelating residues were exactly aligned. Analysis of the ARIH2 sequence using NetSurfP 2.0 predicted residues 66 to 83, 88 to 97, 102 to 111, 113 to 120, 162 to 174, 194 to 200, 204 to 220, 257 to 260, 271 to 277, 281 to 293, 349 to 400, 404 to 434, 440 to 491 to be helical. Residues 66 to 84, 88 to 98, 102 to 110, 113 to 120, 148 to 150, 162 to 172, 194 to 200, 204 to 221, 270 to 281, 361 to 399, 404 to 433, and 438 to 491 were modeled as helical from alignment with ARIH1. ARIH2 residues 281 to 293 were predicted to be part of a longer helix extending from residue 271; however, this segment is shorter in ARIH1 and was therefore modeled as a loop in ARIH2. A similar situation applied to ARIH2 residues 349 to 360. Substitution of ARIH2 for ARIH1 gave the structure of ARIH2-UBE2L3-Ub based on the structure of the complex (PDB 5UDH).

### Docking the N Terminus of ARIH2 into ASB9-CRL

The flexible N terminus of ARIH2 was docked onto ASB9-CRL using GalaxyWEB PepDock (34), CABS-dock (35), and HPEPDOCK (36). These docking programs were selected for their ability to model long peptides (>20 aa) in large proteins (>300 aa) and to discriminately score various models. HPEPDOCK performs blind peptide–protein docking of a given sequence to a submitted structure and returns models with calculated docking energies. PepDock uses template structures of peptide–protein interactions to predict binding interfaces between the given peptide sequence and protein structure and scores the models based on protein and interaction similarities to the template. CABS-dock simulates and clusters trajectories of the given peptide sequence interacting with the submitted protein structure, returning the predominant clusters with RMSD scores, cluster density, and contact maps.

The peptide “MSVDMNSQGSDSNEEDYDPNCEEEEEEEEDDPGDIEDYYVGVASDVEQQGADAFDPE,” corresponding to residues 1 to 57 of ARIH2, was docked onto CUL5(NEDD8)-RBX2, from the post-neddylation homology model, using HPEPDOCK. Ten models were generated, but the structures had little in common with each other and with insignificant contact with CUL5. The peptide “DSNEEDYDPNCEEEEEEEEDDPGDIEDYYV,” corresponding to residues 11 to 40 of ARIH2, was docked onto CUL5_1–780_-RBX2 from the post-neddylation homology model, using PepDock (34). Docking with PepDock produced ten models from four protein–peptide template structures. A particular region of CUL5_CTD_ to which ARIH2 residues 11 to 40 preferentially docked was identified. However, the estimated accuracy score for all of the models was less than 0.4, indicating that less than 40% of the residue interactions in each model were likely to be correct. Furthermore, many of the docked structures seemed improbable because the ARIH2 sequence was threaded through CUL5. In addition, the ARIH2 sequences that were not threaded had contacts with CUL5 that did not match previous mutagenesis.

The peptide “DSNEEDYDPNCEEEEEEEEDDPGDIEDY,” corresponding to residues 11 to 38 of ARIH2 (truncated due to peptide length limitations), was docked onto CUL5_300–780_ (truncated due to protein length limitations), from the post-neddylation homology model, using CABS-dock. Fifty simulation cycles were performed, and ten models were generated. The model with the highest cluster density and no steric clashes with CUL5 was selected as the best model.

### Building ARIH2 onto ASB9-CRL

The best CABS-dock model, consisting of residues 11 to 38 of ARIH2 bound to CUL5, was selected to begin building ARIH2 onto the full ASB9-CRL homology model. Residues 34 to 60 of ARIH2 were modeled using PEP-FOLD3 (37). The model with the lowest sOPEP energy score was chosen. The docked peptide, the PEP-FOLD3 peptide, and the homology model for ARIH2 were used to model ARIH2 onto the ASB9-CRL homology model. MODELLER 9.23 was run using the default automodel class. The model with the lowest MOLPDF score of the ten models generated was selected as the final model.

## Results

### Assembly of an Active ASB9-CRL From Purified Proteins *in Vitro*

Using a process of strategic coexpression and *E. coli* lysate mixing, we were able to assemble the following partial and complete ASB9-CRL protein complexes; CKB-ASB9-ELOB/C-CUL5(±NEDD8)-RBX2, CUL5(NEDD8)-RBX2, ARIH2-CUL5(NEDD8)-RBX2, as well as ARIH2 alone (Fig. 1*B*, supplemental Fig. S1). Neddylation did not require the CKB-ASB9 components and proceeded efficiently by mixing the *E. coli* lysate containing the CUL5 complex, the preformed NAE1/UBA3∼NEDD8 adduct, and the NEDD8 E2, UBE2F with Mg ATP. Full CUL5 neddylation occurred within 30 min at 4 °C (supplemental Fig. S2, see Methods for more details). To assess the thioester transfer of ubiquitin to E2 enzymes (E2∼Ub), we mixed UBE1 and an E2 enzyme with Ub (29) for 3 h at 37 °C and measured band shifts by SDS-PAGE (30) and anti-Ub western blotting. The K724R mutant of CUL5 was used for nonneddylated CUL5 control experiments because we observed that Ub could be attached to CUL5 K724 in the absence of NEDD8 and UBE2F by UBE2D2.

### ARIH2 Promotes CKB Ubiquitylation

**Fig. 2:**
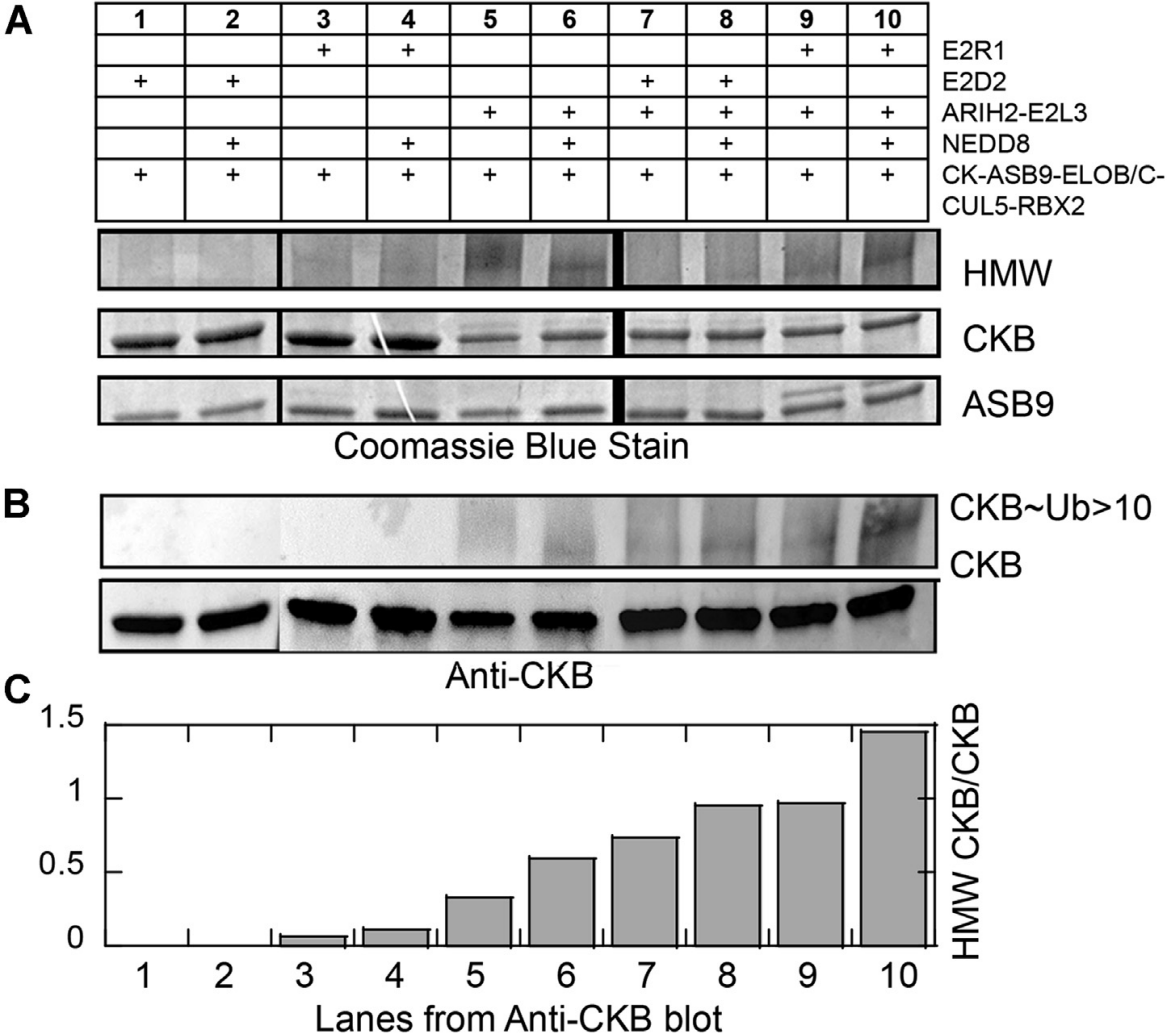
***In vitro* ubiquitylation assays showing AriH2-induced ubiquitylation.***A*, Coomassie Blue–stained gel of various ubiquitylation reactions. All reactions contained MgATP, UBE1, and Ub. Lanes 1, 3, 5, 7, 9 contain CKB-ASB9-ELOB/C-CUL5-RBX2. Lanes 2, 4, 6, 8, 10 contain CKB-ASB9-ELOB/C-CUL5(NEDD8)-RBX2. Lane-specific additional components are UBE2D2 (lane 1, 2), UBE2R1 (lanes 3, 4), ARIH2-UBE2L3 (lanes 5, 6), ARIH2-UBE2L3-UBE2D2 (lanes 7, 8), and ARIH2-UBE2L3-UBE2R1 (lanes 9, 10). *B*, anti-CKB blot for the reactions as in panel *A* showing the increase in CKB migrating at high molecular weight. *C*, image J analysis of the Anti-CKB blot in *B*. The high-molecular-weight CKB ratioed against CKB reveals that the most efficient combination of components for polyubiquitylation of CKB is ARIH2 with UBE2R1. Subsets of this experiment (biological replicates) were repeated three times.

Consistent with a recent report, ARIH2 bound CUL5(NEDD8) with higher affinity compared with un-neddylated CUL5 (supplemental Fig. S3) (22). Addition of ARIH2 and its ubiquitylated E2, UBE2L3∼Ub, resulted in 50% of the CKB being ubiquitylated according to SDS PAGE (Fig. 2*A*). If only UBE2D2 or UBE2R1 was added to CKB-ASB9-ELOB/C-CUL5(+NEDD8)-RBX2, the ratio of CKB to ASB9 remained 2:1 (Fig. 2*A*, lanes 1–4), whereas addition of ARIH2 and UBE2L3∼Ub resulted in migration of one equivalent of the CKB band to higher molecular weight, apparently due to ubiquitylation of one subunit of the dimeric CKB (Fig. 2, *A* and *B* (lanes 5–10). Notably, we never observed migration/ubiquitylation of more than one equivalent of the dimeric CKB across three independent biological replicate experiments, strongly supporting the idea that only one subunit of the CKB dimer is ubiquitylated.

To probe the role of E2 enzymes that bind *via* RBX2, we added either UBE2D2 or UBE2R1 with UBE1 and Ub to the purified ligase (Fig. 2*C*). In three separate biological replicates, we observed that without ARIH2, the ratio of CKB:ASB9 was 1.9 ± 0.3, whereas with ARIH2 (alone or with UBE2D2 or with UBE2R1), the CKB:ASB9 ratio was 1.2 ± 0.4, indicating the migration of one equivalent of the dimeric CKB to higher molecular weight.

To measure ubiquitylation of specific lysines, we took advantage of the fact that trypsin leaves a diglycyl fragment on the lysine that bore the Ub that is readily detectable by MS/MS sequencing (33). The *in vitro* ubiquitylation samples were acetylated and analyzed by MS/MS. After trypsin digestion, approximately 200 peptides were observed from CKB that covered 93% of the CKB sequence and all but one (K319) of the 18 lysines. By comparing the total intensity of acetylated peptide *versus* ubiquitylated peptide, we could obtain information about the likelihood that each lysine was ubiquitylated. Remarkably, very few of the 18 lysines were observed to be ubiquitylated (supplemental Table S1). The most highly modified sites were K45 and K381, but K101 and K107 were also modified. MS/MS analysis also revealed which lysines of ubiquitin were linked. The ligase with ARIH2 either alone or with UBE2D2 or UBE2R1 produced a mixture of K48 and K63 Ub chain linkages on the same four lysines (supplemental Table S2). MS/MS data collected on the high-molecular-weight species (*cf.* Fig. 2*C*) showed only CKB and Ub in different ratios with the highest ratio of Ub to CKB present when ARIH2 and UBE2R1 were present, suggesting that the combination of ARH2 and UBE2R1 generated the most polyubiquitylation.

### Models of the Structures of the Neddylated ASB9-CRL With ARIH2 Bound

Using a combination of cryoEM data and homology modeling, we previously obtained a model of the CKB-ASB9-ELOB/C-CUL5-RBX2 complex in which the CUL5 was not neddylated (16). Here, we used homology modeling to generate the structure of the CUL5_CTD_(NEDD8)-RBX2 based on the structure of CUL5_CTD_(NEDD8)-RBX1 (PDB 3DQV), by aligning residues 208 to 238 of RNF4 in RNF4-UBE2D1-Ub (4AP4) to RBX1 to position UBE2D1-Ub. Finally, RBX1 was replaced with RBX2. The model has UBE2D1, which is very similar to UBE2D2 (89% identity, 97% similarity), and both E2s functioned in our ubiquitylation assays.

**Fig. 3:**
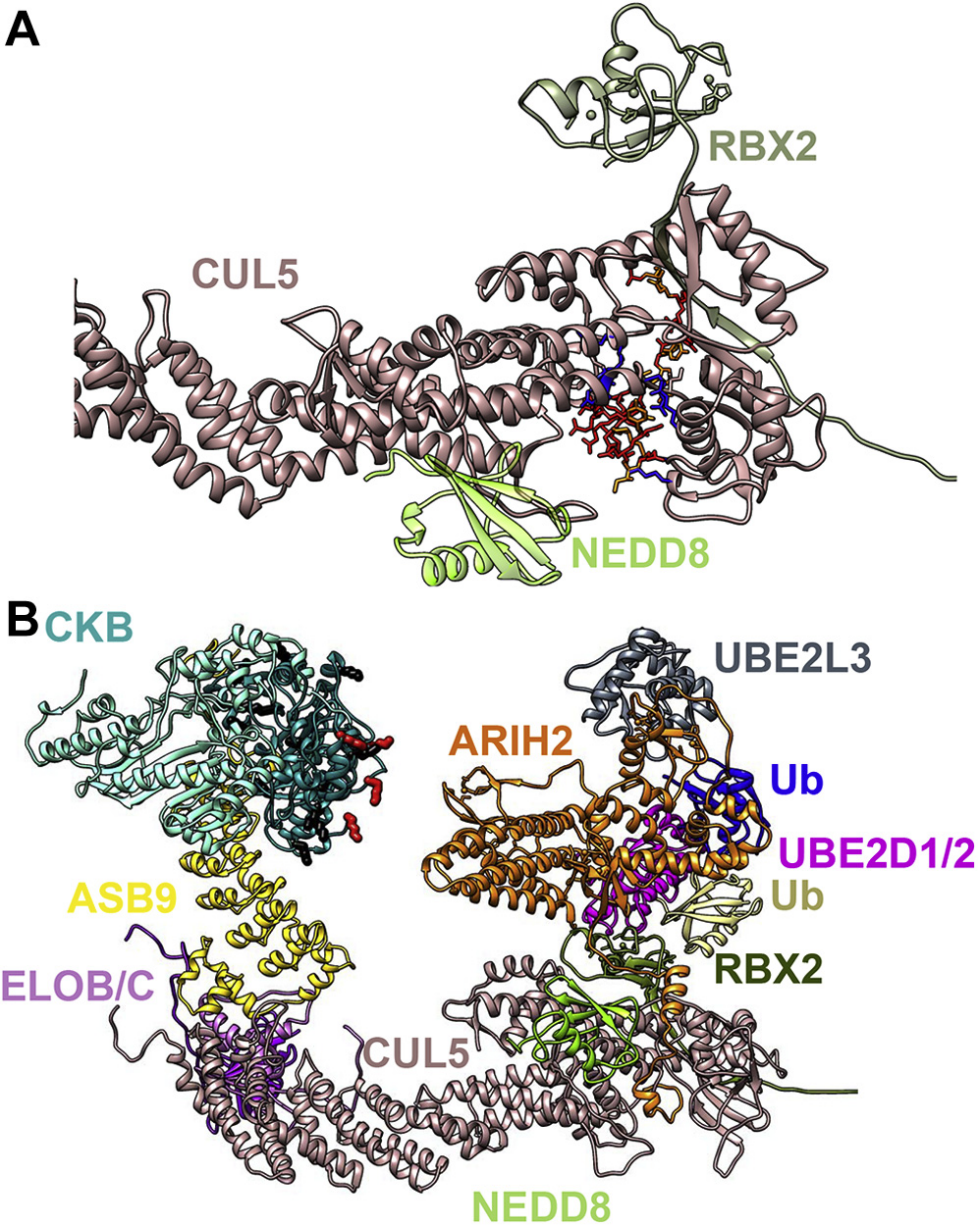
**Model of the ASB9-CRL showing sites of ubiquitylation on CKB.***A*, molecular docking was used to place ARIH2 residues 11 to 38 into the basic cleft in CUL5. Acidic residues on ARIH2 are shown in *red* and CUL5 basic residues previously shown to be critical for the interaction (21) are shown in *blue*. *B*, a structural model of CKB-ASB9-ELOB/C-CUL5(NEDD8)-RBX2-E2D1/2∼Ub-ARIH2-UBE2F∼Ub built from the published structure of ASB9-ELOB/C-CUL5-RBX2-UBE2D1/2 (16) by addition of ARIH2-UBE2F∼Ub based on the docked structure from *A*). Homologous structural information is not available until residue 57 of ARIH2, so the absolute position of ARIH2 relative to the ASB9-CRL is speculative. The four CKB lysines (K45, K101, K107, and K381) on one subunit of the monomeric CKB that were observed to be ubiquitylated (*red side chains*) are the closest lysines to ARIH2. The protein colors are consistent throughout the manuscript: CKB, *aquamarine*, *dark cyan*; ASB9, *yellow*; ELOB/C, *orchid*, *purple*; CUL5, *rosy brown*, RBX2, *olive*; NEDD8, *chartreuse*; UBE2D1/2, *magenta*; Ub on UBE2D1/2, *khaki*; ARIH2, *orange*, UBE2L3, *gray*; Ub on UBE2F, *blue*.

A homology model of ARIH2 was built based on the structure of ARIH1 (5UDH) by prioritizing the residues that coordinate the two zinc atoms in the RING and in-between-RING domains (supplemental Fig. S4). The interaction of ARIH2 with CUL5 was shown by mutagenesis to depend on residues R417, K418, K423, K424, K676, K679, K682, and R683 (21). Therefore, we used CABS-DOCK to dock the acidic N-terminal residues 11 to 38 of ARIH2 to this basic patch on CUL5 (35) (Fig. 3*A*). ARIH2 contains additional residues (34–60) not present in ARIH1, which were modeled to complete the predicted structure of ARIH2, which was then assembled with CKB-ASB9-ELOB/C-CUL5(NEDD8)-RBX2. The final model of the ARIH2-bound to the ASB9 CRL showing the ubiquitylated CKB residues K45, K101, K107, and K381 is shown in Figure 3*B*. The four lysines that we found to be ubiquitylated appear to be the CKB surface lysines closest to the ARIH2. In line with our observation that only one subunit of CKB gets modified (see above), one subunit of CKB is much closer to ARIH2 than the other.

### Both ARIH2 and RBX2 Appear to Be Flexibly Tethered to CUL5

**Fig. 4:**
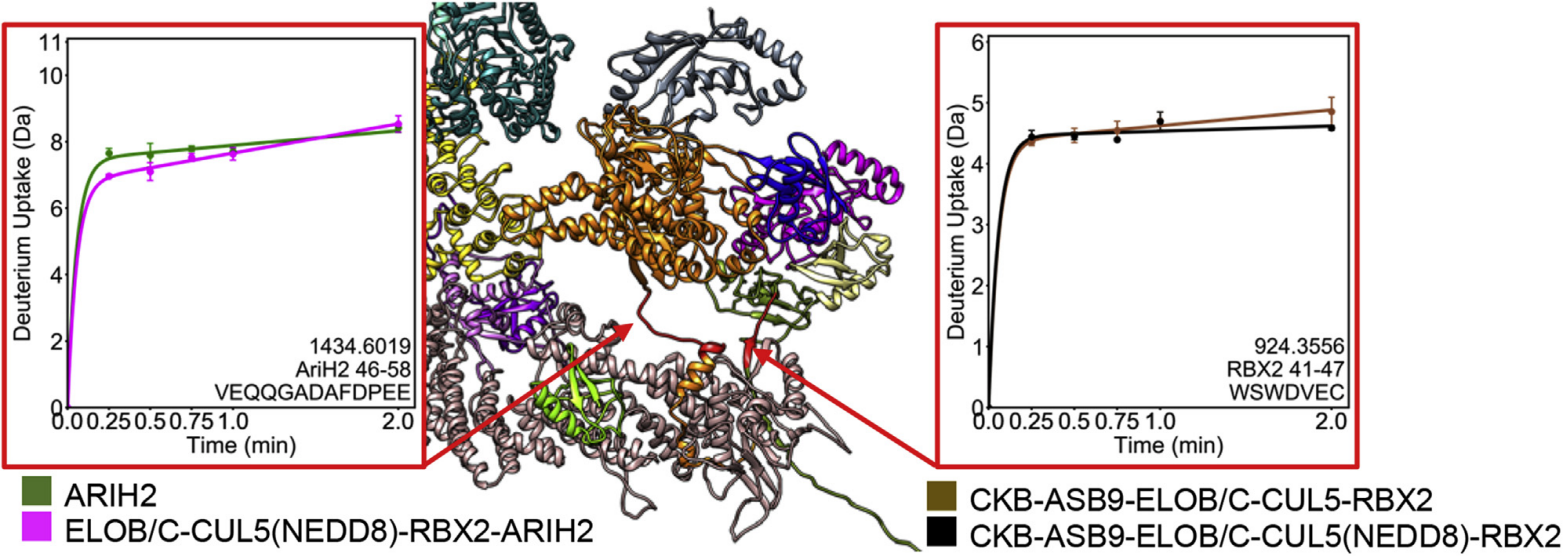
**HDX-MS reveals flexible tethers on ARIH2 and RBX2.** HDX-MS shows residues 46 to 58 of ARIH2 are highly exchanging when ARIH2 is alone (*green*) or bound to the ASB9-CRL (*magenta*). This segment of ARIH2 (*orange*) is colored in *red* in the structural model of the ASB9-CRL (expanded view). Similarly, RBX2 residues 41 to 47 are highly exchanging in the ASB9-ELOB/C-CUL5-RBX2 complex (*brown*) as well as in the neddylated complex (*black*). This segment of RBX2 (*olive*) is also colored red in the structural model of the ASB9-CRL (expanded view).

Both ARIH2 and RBX2 have segments that are predicted to be unstructured that tether them to CUL5. HDX-MS measurements of deuterium uptake revealed that these apparent tethers, residues 46 to 58 of ARIH2 and residues 41 to 47 of RBX2, are highly exchanging even after only 30 s of exchange (Fig. 4) consistent with a lack of structure. These tethers are likely to be important for optimal orientation for transfer of the Ub, which would be attached to either ARIH2 or to one of the other E2s bound to RBX2.

### Neddylation Restructures CUL5 and RBX2

**Fig. 5:**
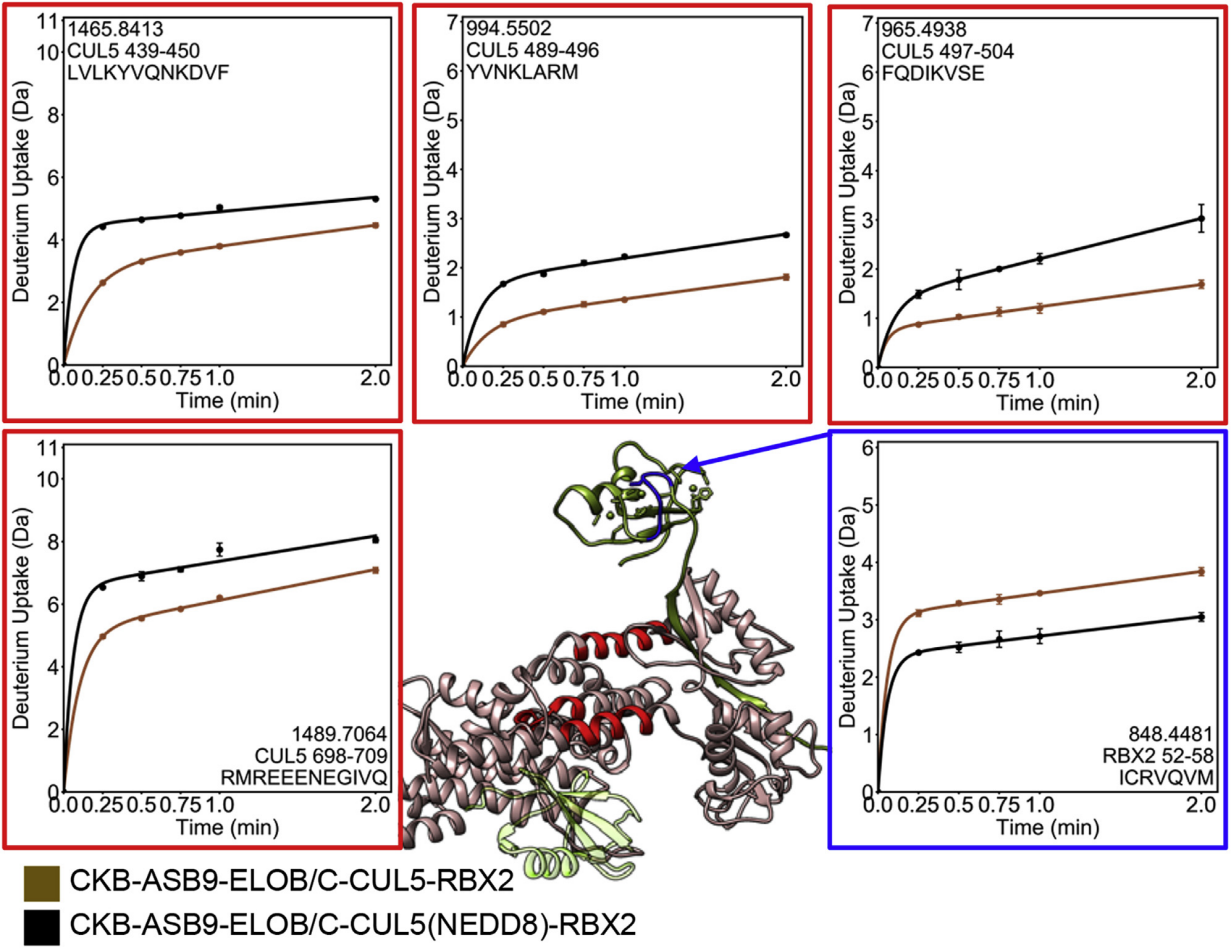
**HDX-MS reveals long-range allostery upon neddylation of CUL5.** Comparison of the HDX-MS deuterium uptake into CKB-ASB9-ELOB/C-CUL5-RBX2 complex (*brown symbols* in plots) with the CKB-ASB9-ELOB/C-CUL5(NEDD8)-RBX2 complex (*black symbols* in plots) revealed that neddylation of CUL5 causes increased exchange in CUL5 residues (*red*) and decreased exchange in RBX2 residues (*blue*).

Neddylation of CUL5 is known to play an important role in the activation and regulation of CUL-RING ligases (38). Crystal structures of the C-terminal domain of CUL5 before and after neddylation show that the modification reorients the C-terminal domain of CUL5 and increases the angle between CUL5 and RBX1 (3DPL, 3DQV). We performed HDX-MS experiments on CKB-ASB9-ELOB/C-CUL5-RBX2 and CKB-ASB9-ELOB/C-CUL5(NEDD8)-RBX2 to discover possible long-range conformational alterations that may occur upon neddylation. When CUL5 was neddylated, CUL5 residues 439 to 450, 489 to 496, 497 to 504, and 698 to 709 exchanged more, implying increased dynamics and/or solvent exposure of these regions (Fig. 5, supplemental Fig. S6). We note that CUL5 neddylation did not cause any observable decreases in exchange. Only 82% of the CUL5 sequence was covered in our HDX-MS data and residues 711 to 725 were not covered, so it is possible that NEDD8 contacts the surface of CUL5 near its attachment site. A completely unexpected decrease in exchange of RBX2 residues 52 to 58 was observed when neddylated CUL5-RBX2 was compared with unneddylated CUL5-RBX2. This RBX2 sequence is solvent exposed in the homology models both before and after neddylation. This region contains Arg 54, which by analogy with RBX1 is the linchpin residue (Arg 46 in RBX1), which is inserted between the NEDD8 and its E2 in the structure of the analogous proteins (PDB code 4P50, supplemental Fig. S5).

### ARIH2 Binding to the ASB9-CRL Causes Decreased Exchange in CUL5 and Significantly Increased Exchange in ARIH2

**Fig. 6:**
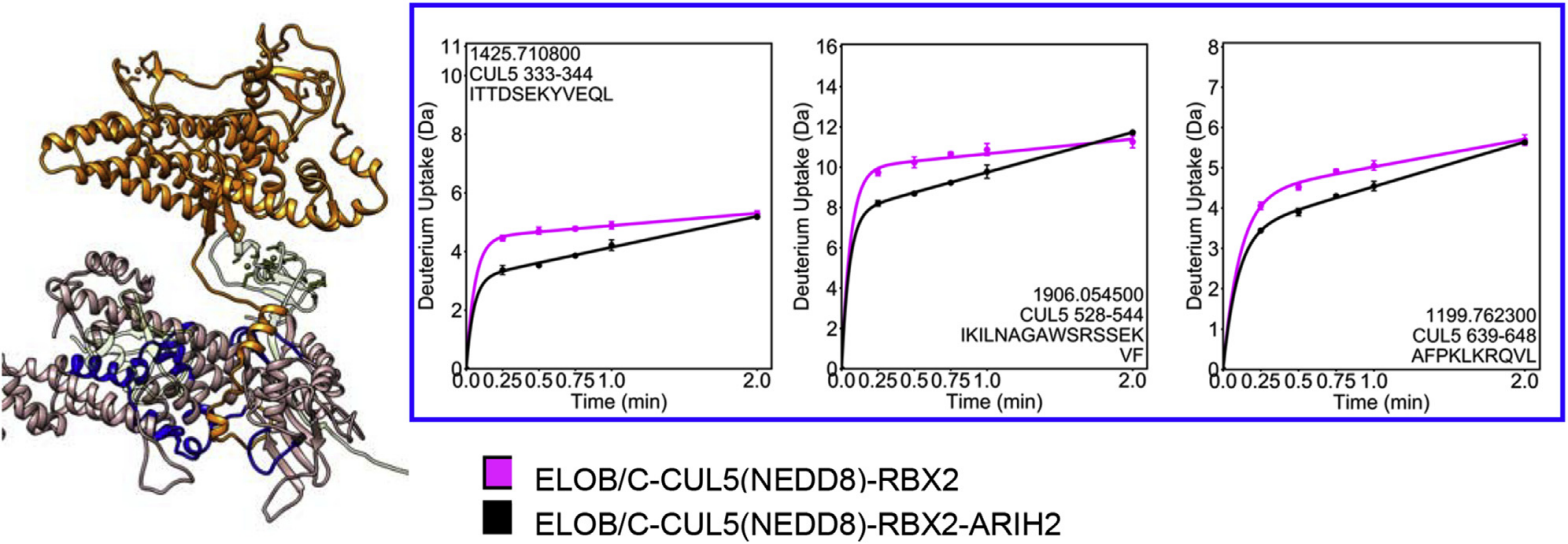
**HDX-MS reveals transient protection of CUL5 by the N terminus of ARIH2.** Comparison of the HDX-MS deuterium uptake into ELOB/C-CUL5(NEDD8)-RBX2 complex (*magenta* symbols in plots) with the ELOB/C-CUL5(NEDD8)-RBX2-ARIH2 complex (*black* symbols in plots) revealed that the CUL5 regions that form the basic cleft and the surrounding residues are transiently protected upon ARIH2 binding (*blue* regions in structure).

Next, we compared free ARIH2 and free ELOB/C-CUL5(NEDD8)-RBX2 with the complex between them (ELOB/C-CUL5(NEDD8)-RBX2-ARIH2) by HDX-MS. The ARIH2 peptides containing the highly acidic residues that our docking (and the published mutagenesis experiments (21)) predict should interact with the positively charged patch on the C terminus of CUL5 were not detected in the HDX-MS experiments. We did not identify any other surface of ARIH2 that showed decreased exchange upon binding to the CRL. However, comparison of deuterium uptake into the ELOB/C-CUL5(NEDD8)-RBX2 and ELOB/C-CUL5(NEDD8)-RBX2-ARIH2 complexes revealed CUL5 residues 333 to 344, 366 to 383, 415 to 429, 439 to 450, 465 to 471, 477 to 488, 528 to 544, and 639 to 648 decreased exchange in the presence of ARIH2. Interestingly, this effect was transient, disappearing by the 2 min timepoint (Fig. 6, supplemental Fig. S7). We recall that neddylation caused increased exchange in CUL5 at regions 439 to 450 and 489 to 504, at or between the same areas that then decreased exchange upon ARIH2 binding. The protection of these regions of CUL5 upon ARIH2 binding is consistent with our docked model, which places ARIH2 residues 11 to 38 in this cleft on CUL5 that is opened up by neddylation.

**Fig. 7:**
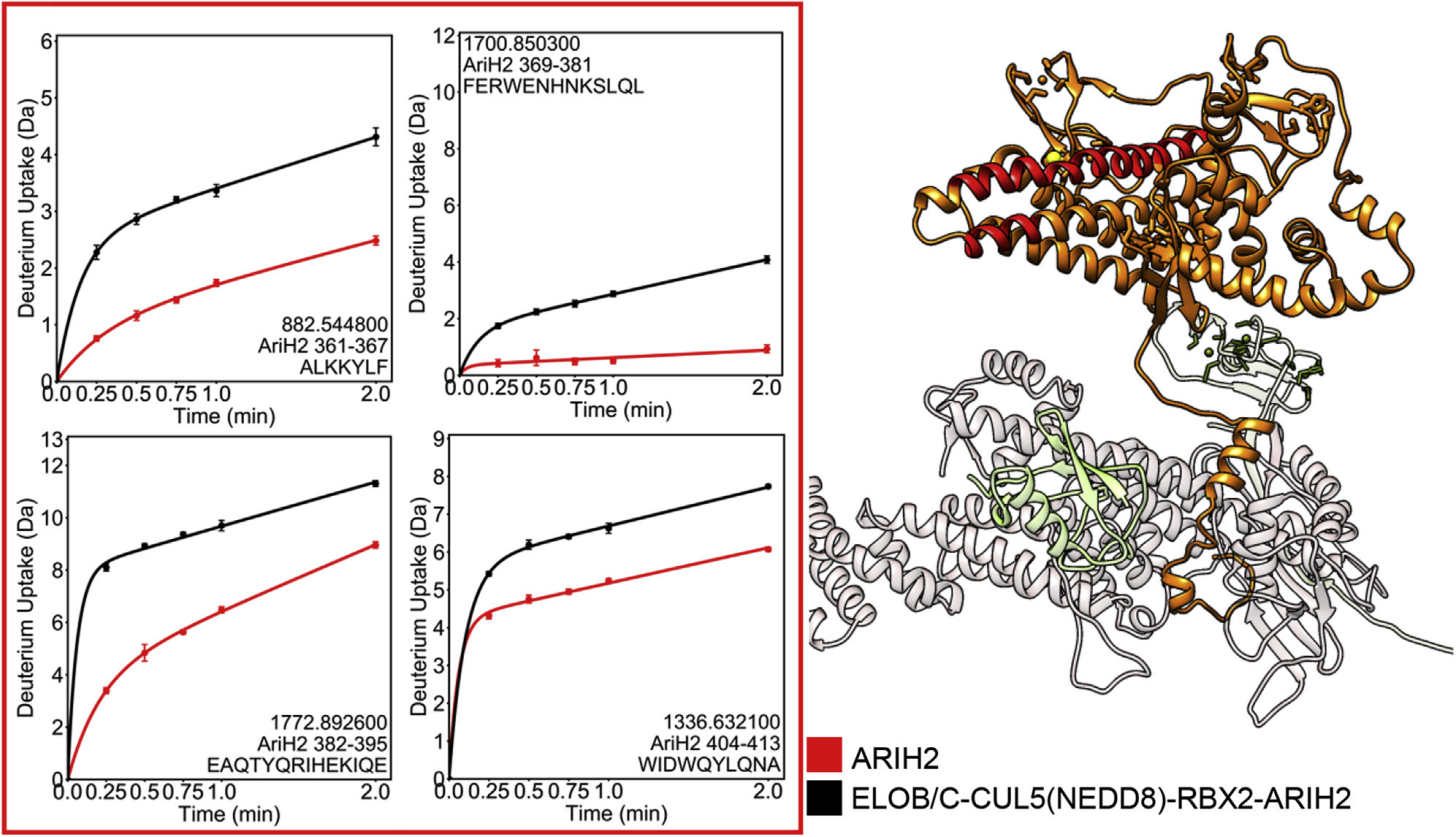
**HDX-MS of AriH2 reveals the mechanism for relief of autoinhibition.** Comparison of the HDX-MS deuterium uptake into ARIH2 alone (*red symbols* in the plots) *versus* in complex with the ASB9-CRL (*black symbols* in the plots) shows marked increase in exchange (*red regions* in the structure) of the helical regions surrounding the ARIH2 active site upon binding to ASB9-CRL.

Finally, several regions of ELOB/C-CUL5(NEDD8)-RBX2-bound ARIH2 (residues 361–367, 369–381, 382–395, 404–413) showed marked increases in deuterium exchange compared with free ARIH2 (Fig. 7, supplemental Fig. S8). These regions are near the catalytic cysteine and surround the domain of ARIH2 that has been previously reported to be autoinhibited prior to CUL5 binding (21). These results strongly indicate a long-range allosteric mechanism by which ARIH2 autoinhibition is released upon interaction with CUL5, which involves increased dynamics/exposure of these ARIH2 regions, which surround the ARIH2 active site.

## Discussion

### Activity and Specificity of the ASB CRL

We were able to reconstruct the full ASB9-CRL *in vitro* from recombinant proteins purified from *E. coli* and demonstrate efficient CKB ubiquitylation. The CUL5-RBX2 was readily neddylated in the absence of the substrate receptor (ASB9) and substrate (CKB) suggesting that substrate engagement is not required for CRL neddylation. ARIH2 was found to be critical for high-efficiency ubiquitylation of the substrate, CKB. Indeed, ARIH2 is essential for ubiquitylation of a number of important substrates, such as APOBEC3 by the CUL5^Vif/CBFß^ E3 ligase (22). Although others have suggested that ARIH2-UBE2L3 only installs the first Ub and then the polyubiquitin chain is elongated by the E2 bound to RBX2 (20), our results show that ARIH2-UBE2L3∼Ub bound with high affinity to the neddylated CRL and rapidly polyubiquitylated one equivalent of the CKB dimer at four specific lysines creating both K48- and K63-linked polyubiquitin chains on CKB. Addition of UBE2R1 or UBE2D2 supplemented ubiquitylation, but these E2s could not efficiently ubiquitylate CKB in the absence of ARIH2.

### Structural Model of the ARIH2-Bound ASB-CRL

Based on a previous structure of the ASB9-CRL (16) and previous mutagenesis experiments (21), ARIH2 was docked to form a predicted structure of the full neddylated ASB9-CRL with ARIH2 bound. HDX-MS experiments identified several regions of CUL5 that increased in exchange upon neddylation and then decreased in exchange upon ARIH2 binding strongly suggesting a mechanism for how neddylation promotes ARIH2 binding (22). The observation that the decreased exchange in the CUL5_CTD_ was transient for all regions suggests that even though the affinity of ARIH2 for CUL5 is high enough to observe co-elution in SEC, the interacting residues continue to exchange with solvent over time. This may be a function of the highly charged nature of the interaction.

The structural models and HDX-MS results show that both ARIH2 and RBX2 are attached to CUL5 by flexible tethers, which could allow them to be in multiple locations further accommodating simultaneous binding and/or Ub transfer once the first Ub is attached to CKB by ARIH2.

### Functional Dynamics and Allostery in the ASB CRL

Comparison of several states of the ASB9-CRL revealed differences in H/D exchange indicative of long-range allosteric mechanisms that activate the ligase. First, it is known that RBX2 associates with UBE2F for neddylation (39) of CUL5 at K724. Once this is accomplished, RBX2 needs to release UBE2F and bind a ubiquitylating E2. We found that CUL5 neddylation triggered decreased exchange in RBX2 at Arg54. By analogy with RBX1, this is the “linchpin” residue that inserts between the NEDD8 and its E2 in the structure of the analogous proteins (PDB code 4P50) (39). These results suggest that CUL5 neddylation may induce long-range conformational alterations in RBX2 to discourage binding of the NEDD8 E2 to RBX2. These HDX results suggest a long-range allosteric mechanism by which the specificity of RBX2 for different E2 enzymes changes following neddylation (40). This long-range allostery would need to pass through the ostensibly flexible tether linking RBX2 to CUL5. This type of allostery has been seen before in at least one other multidomain protein (27). We find that the model where CRL may hinge in order to allow Ub transfer across the >60 Å distance between Ub and the substrate is insufficient to explain the ubiquitylation of CKB by the ASB9-CRL. Instead, for substrates that are far away from the RBX2-linked E2∼Ub, the RBRL ARIH2-UBE2L3∼Ub is required.

Second, we found that although neddylation did not alter the substrate-receptor side of the ligase, it increased the solvent exposure of the cleft where ARIH2 binds. This observation suggests that CUL5 is autoinhibited for high-affinity ARIH2 binding until it is neddylated. NEDD8 attachment allosterically opens the CUL5 cleft into which ARIH2 binds, strengthening the ARIH2 binding affinity. ARIH2-UBE2L3 was necessary and sufficient for high-efficiency poly-ubiquitylation of the substrate, CKB, which binds to the N-terminal end of ASB9 and would be too far from the RBX2-E2∼Ub, at least initially.

Third, comparison of free ARIH2 and the ASB9-CRL-ARIH2 complex revealed that the interaction between ARIH2 and CUL5 markedly increased the dynamics of regions of the Ariadne domain surrounding the ARIH2 active site. It is interesting to speculate that somehow the UBE2L3∼Ub can only be transferred to C310 in the Ariadne domain of ARIH2, once this region is opened when the ARIH2 engages with CUL5. Binding of the ARIH2 N-terminal acidic sequence to the CUL5 basic cleft apparently induces a long-range allosteric mechanism, which releases the ARIH2 autoinhibition, presumably by increasing access to the catalytic cysteine. All of these allosteric changes allow ARIH2 to ubiquitylate substrates on the ASB9-CRL.

## Conclusions

**Fig. 8:**
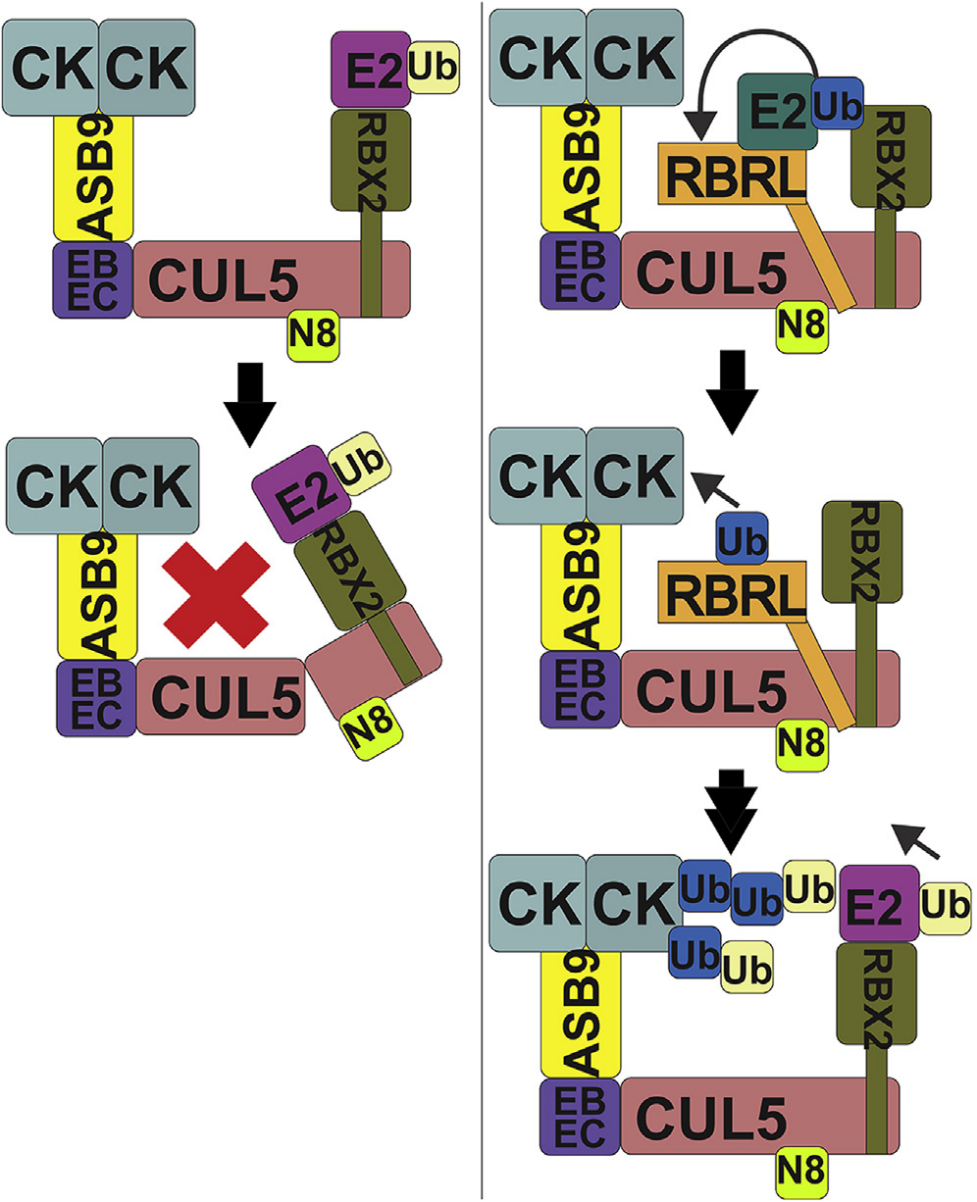
**Schematic diagram of the CRL E3 ligase mechanism based on the results of this study.** Our data support a mechanism by which RBX2 initially engages UBE2F to neddylate CUL5. Neddylation alters the RBX2 to decrease exchange at its binding site for UBE2F. Neddylation also alters the conformation of CUL5 opening a cleft of basic residues to which the acidic C terminus of ARIH2 can bind with high affinity. ARIH2 is autoinhibited, but upon binding to CUL5, its active site increases exchange *via* a long-range allosteric mechanism. The increased exchange likely indicates opening of the ARIH2 active site and relief of autoinhibition. After initial Ub transfer by ARIH2 to specific lysines on CKB that are close to the ARIH2 active site, polyubiquitylation on those same lysines can occur by a number of different routes including continued ubiquitylation by ARIH2 or contributions from UBE2D2 or UBE2R1 bound to RBX2.

By comparing *in vitro* activity, structures, and HDX-MS dynamics of the ASB9-CRL, we were able to answer key mechanistic questions and propose a model of how neddylation of CUL5 E3 ligases activates them for ubiquitylation (Fig. 8). 1) Neddylation of CUL5 allosterically signals to the CUL5-bound RBX2 that the ligase is already neddylated by discouraging its binding to another UBE2F, while allowing its binding of UBE2D2 or UBE2R1 to ubiquitylate substrates. 2) Neddylation of CUL5 allosterically opens the channel into which the N-terminal disordered acidic segment of ARIH2 binds. 3) ARIH2 binding to CUL5 allosterically opens the ARIH2 active site releasing its autoinhibition. ARIH2 efficiently ubiquitylates CKB at specific lysines that are the closest ones to the ARIH2 active site. 4) Polyubiquitylation proceeds after initial ubiquitylation by ARIH2-UBE2L3∼Ub. Addition of UBE2D2 or UBE2R1 to RBX2 is not required for polyubiquitylation *in vitro*, but either E2 can apparently augment the ARIH2 polyubiquitylation. The flexible tethers that connect ARIH2 and RBX2 to CUL5 may allow the additional RBX2-E2∼Ub to reach the already ubiquitylated substrate lysines to extend the poly-ubiquitin chains.

## Data Availability

In the Supporting Material, we have provided all of the State Data Excel files that can be used to generate all of the uptake plots for all of the proteins and complexes reported here. To generate uptake plots automatically from state data, it is best to use DECA (https://github.com/komiveslab/DECA). MS/MS data for ubiquitylation including annotated spectra and all HDX-MS data are available at massive.ucsd.edu under data set MSV000084812.

## Conflicts of interest

Authors declare no competing interests.

## Supplementary Data

MCPRO_MCP/2020/002414_1

MCPRO_MCP/2020/002414_2

MCPRO_MCP/2020/002414_3
