# Supplementary material for: The Mechanism of NEDD8 Activation of CUL5 Ubiquitin E3 Ligases
Source: Mol Cell Proteomics. 2021 Jan 6;20:100019. doi: 10.1074/mcp.RA120.002414 (PMC7950132; doi:10.1074/mcp.RA120.002414)
Supplement: MCPRO_MCP/2020/002414_3 [file mmc3.pptx]

## Slide 1
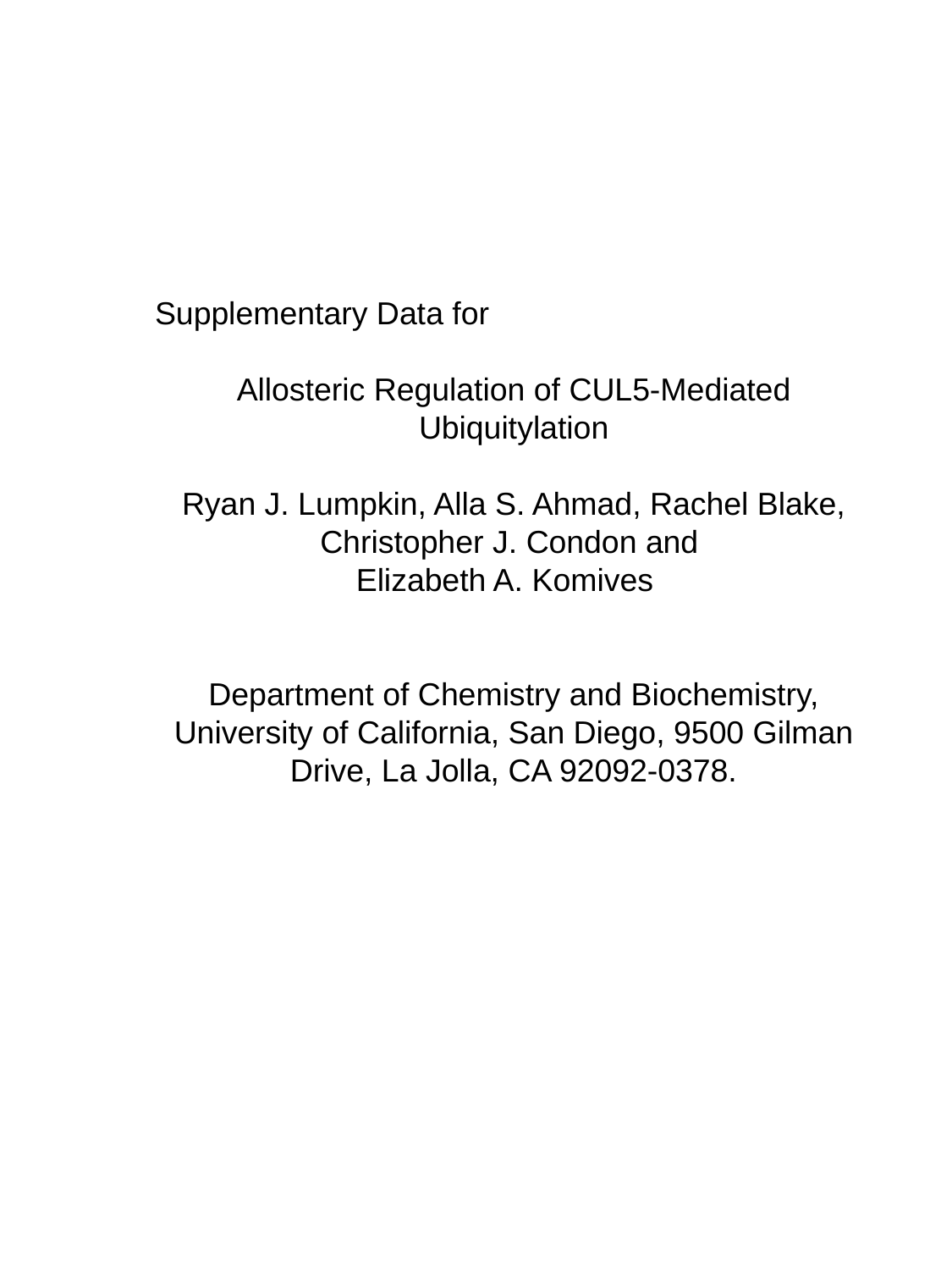

Supplementary Data for
Allosteric Regulation of CUL5-Mediated Ubiquitylation
Ryan J. Lumpkin, Alla S. Ahmad, Rachel Blake, Christopher J. Condon and
Elizabeth A. Komives
Department of Chemistry and Biochemistry, University of California, San Diego, 9500 Gilman Drive, La Jolla, CA 92092-0378.

## Slide 2
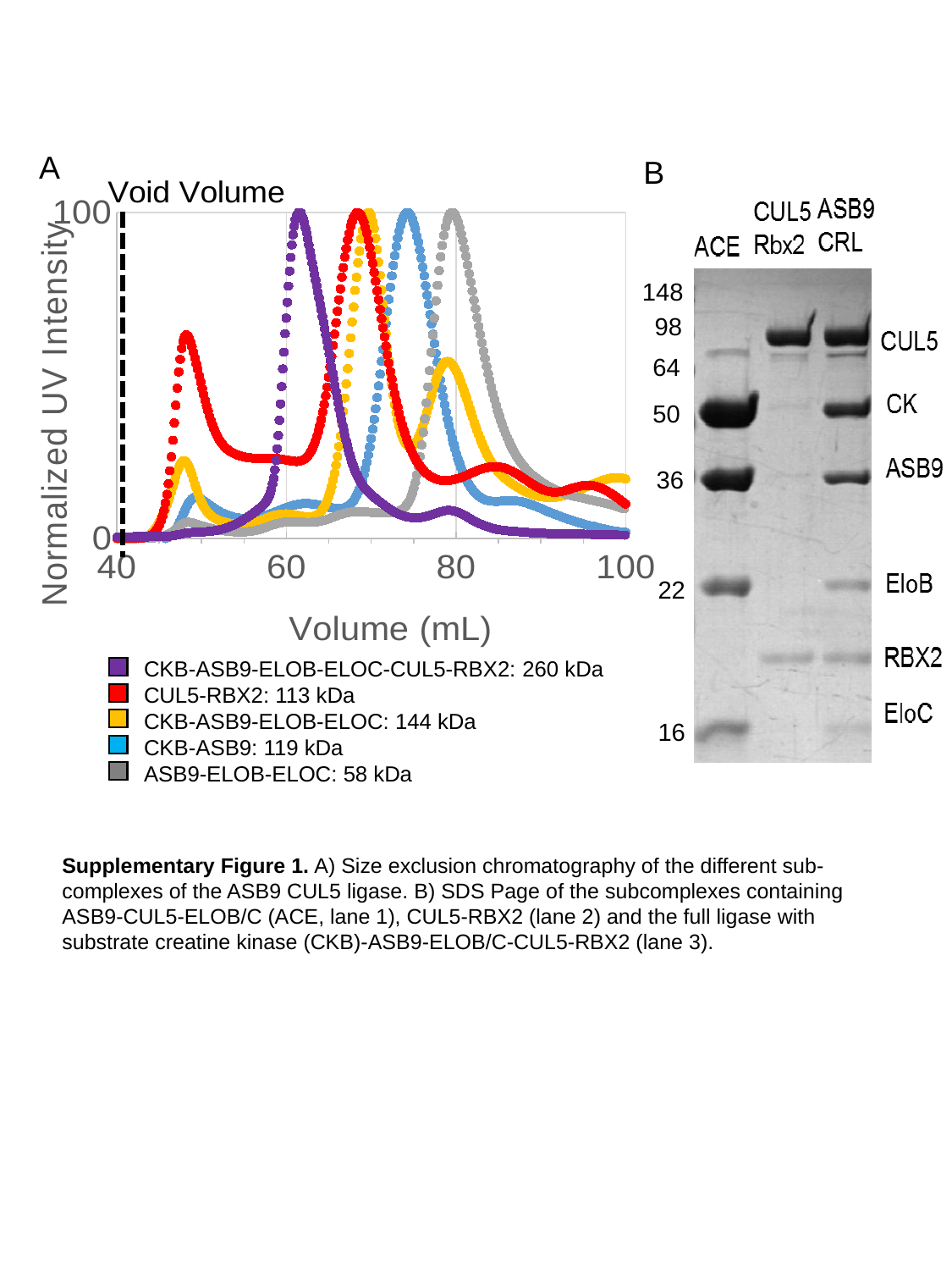

A
B
### Chart
| Category | | | | | |
|---|---|---|---|---|---|
148
98
64
50
36
22
16
CKB-ASB9-ELOB-ELOC-CUL5-RBX2: 260 kDa
CUL5-RBX2: 113 kDa
CKB-ASB9-ELOB-ELOC: 144 kDa
CKB-ASB9: 119 kDa
ASB9-ELOB-ELOC: 58 kDa
Supplementary Figure 1. A) Size exclusion chromatography of the different sub-complexes of the ASB9 CUL5 ligase. B) SDS Page of the subcomplexes containing ASB9-CUL5-ELOB/C (ACE, lane 1), CUL5-RBX2 (lane 2) and the full ligase with substrate creatine kinase (CKB)-ASB9-ELOB/C-CUL5-RBX2 (lane 3).

## Slide 3
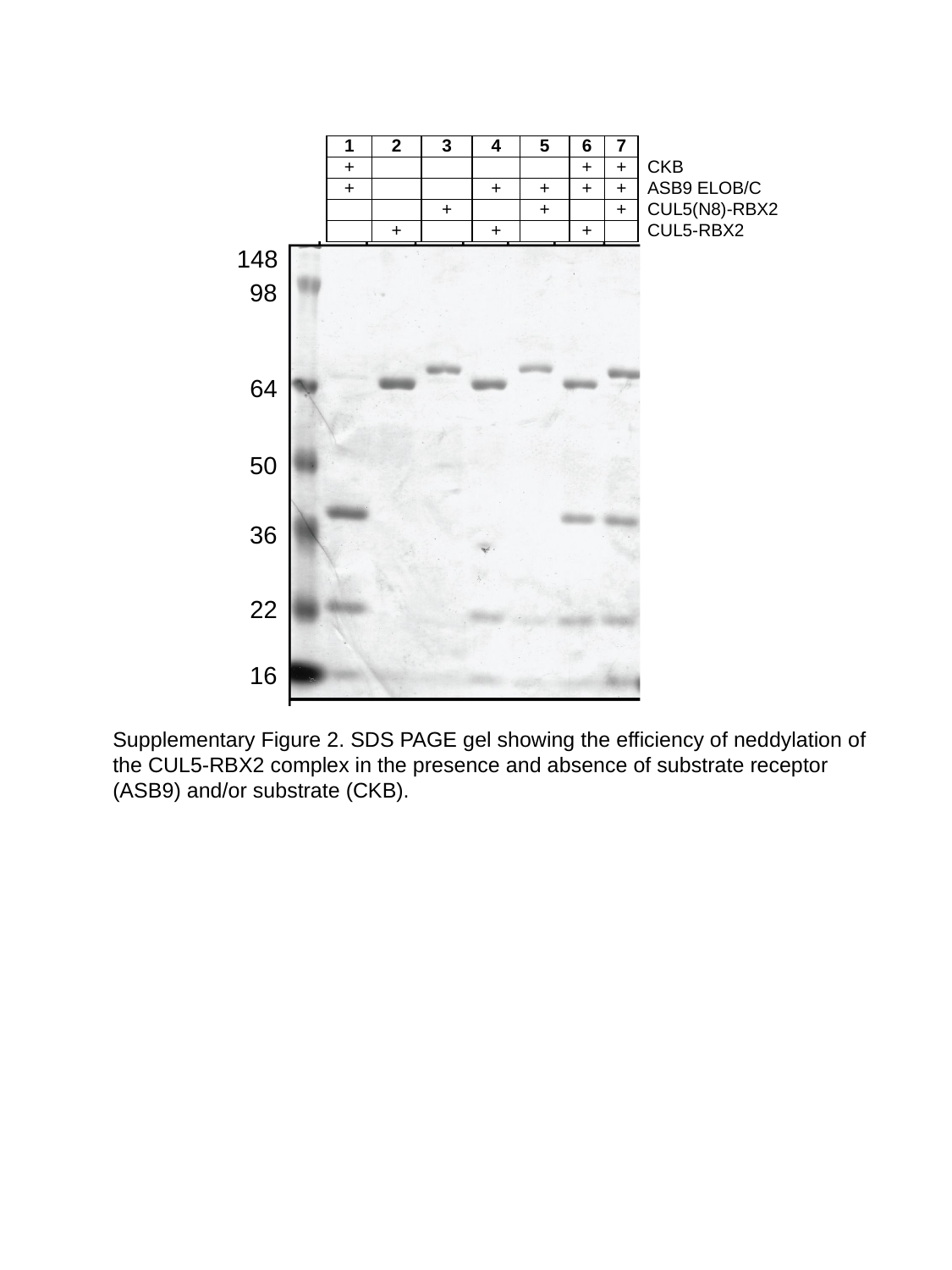

| 1 | 2 | 3 | 4 | 5 | 6 | 7 | |
| --- | --- | --- | --- | --- | --- | --- | --- |
| + | | | | | + | + | CKB |
| + | | | + | + | + | + | ASB9 ELOB/C |
| | | + | | + | | + | CUL5(N8)-RBX2 |
| | + | | + | | + | | CUL5-RBX2 |
148
98
64
50
36
22
16
Supplementary Figure 2. SDS PAGE gel showing the efficiency of neddylation of the CUL5-RBX2 complex in the presence and absence of substrate receptor (ASB9) and/or substrate (CKB).

## Slide 4
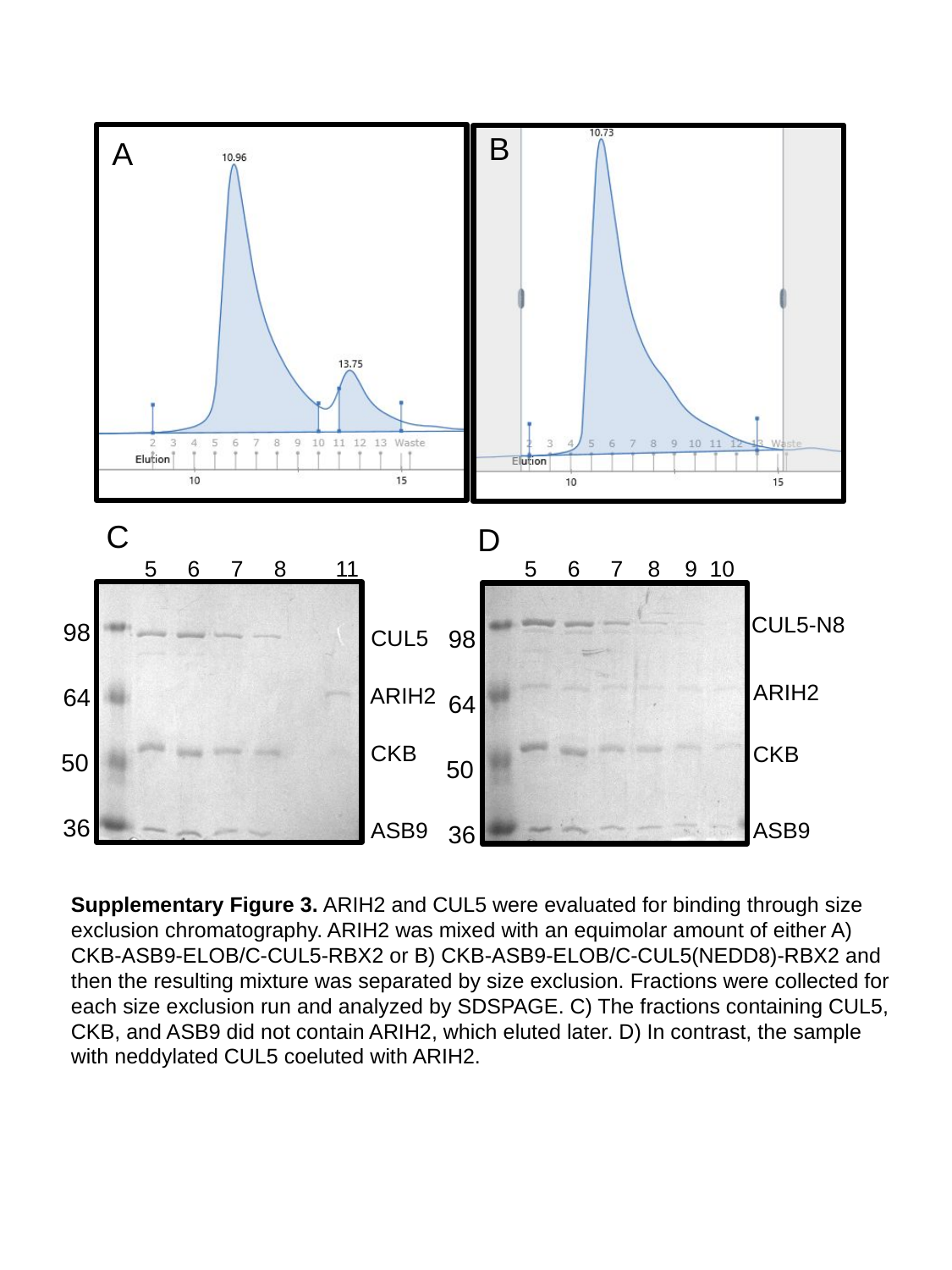

B
A
C
D
5 6 7 8 11
5 6 7 8 9 10
98
64
50
36
98
64
50
36
CUL5-N8
CUL5
ARIH2
ARIH2
CKB
CKB
ASB9
ASB9
Supplementary Figure 3. ARIH2 and CUL5 were evaluated for binding through size exclusion chromatography. ARIH2 was mixed with an equimolar amount of either A) CKB-ASB9-ELOB/C-CUL5-RBX2 or B) CKB-ASB9-ELOB/C-CUL5(NEDD8)-RBX2 and then the resulting mixture was separated by size exclusion. Fractions were collected for each size exclusion run and analyzed by SDSPAGE. C) The fractions containing CUL5, CKB, and ASB9 did not contain ARIH2, which eluted later. D) In contrast, the sample with neddylated CUL5 coeluted with ARIH2.

## Slide 5
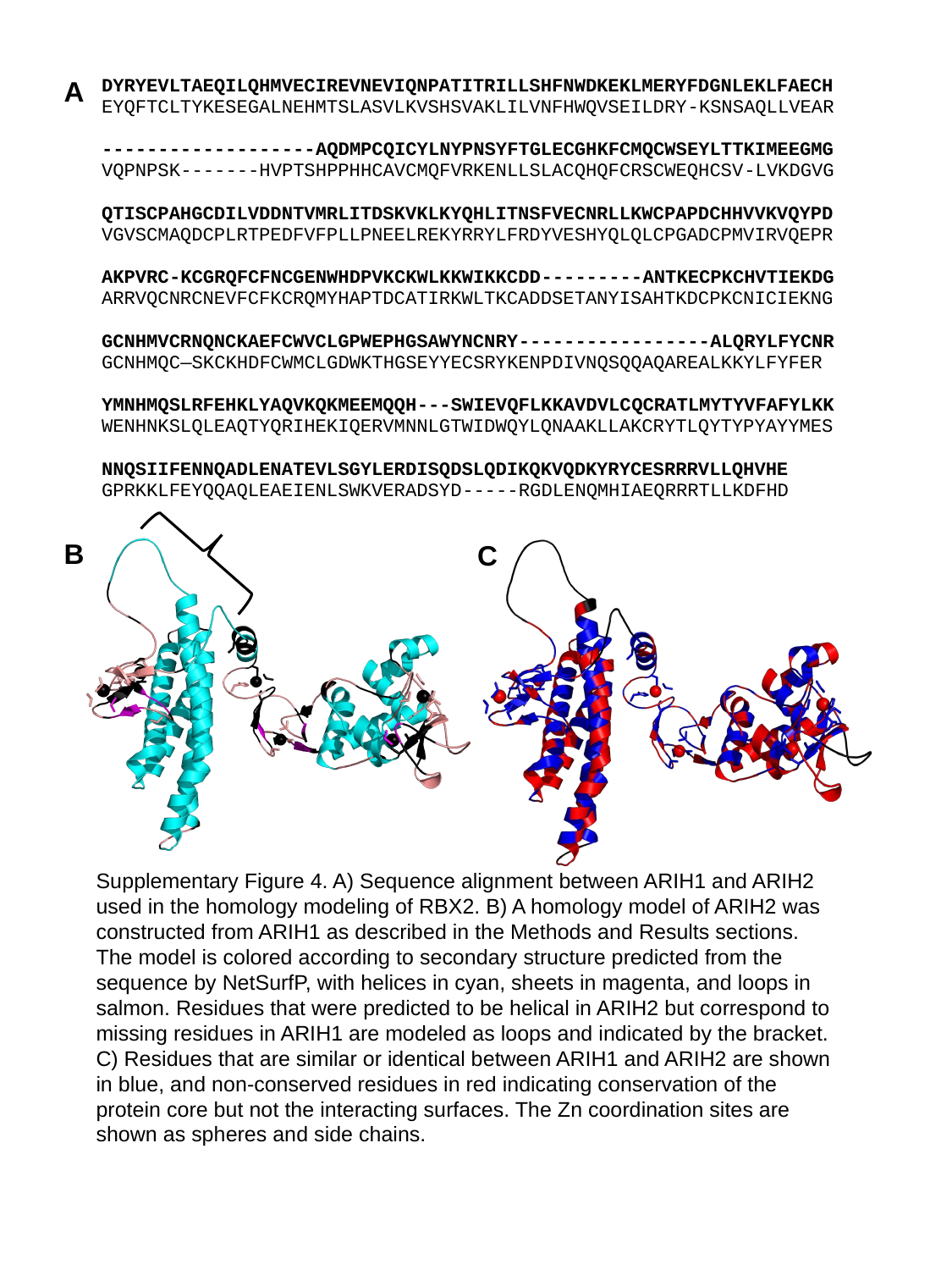

A
B
C
Supplementary Figure 4. A) Sequence alignment between ARIH1 and ARIH2 used in the homology modeling of RBX2. B) A homology model of ARIH2 was constructed from ARIH1 as described in the Methods and Results sections. The model is colored according to secondary structure predicted from the sequence by NetSurfP, with helices in cyan, sheets in magenta, and loops in salmon. Residues that were predicted to be helical in ARIH2 but correspond to missing residues in ARIH1 are modeled as loops and indicated by the bracket. C) Residues that are similar or identical between ARIH1 and ARIH2 are shown in blue, and non-conserved residues in red indicating conservation of the protein core but not the interacting surfaces. The Zn coordination sites are shown as spheres and side chains.

## Slide 6
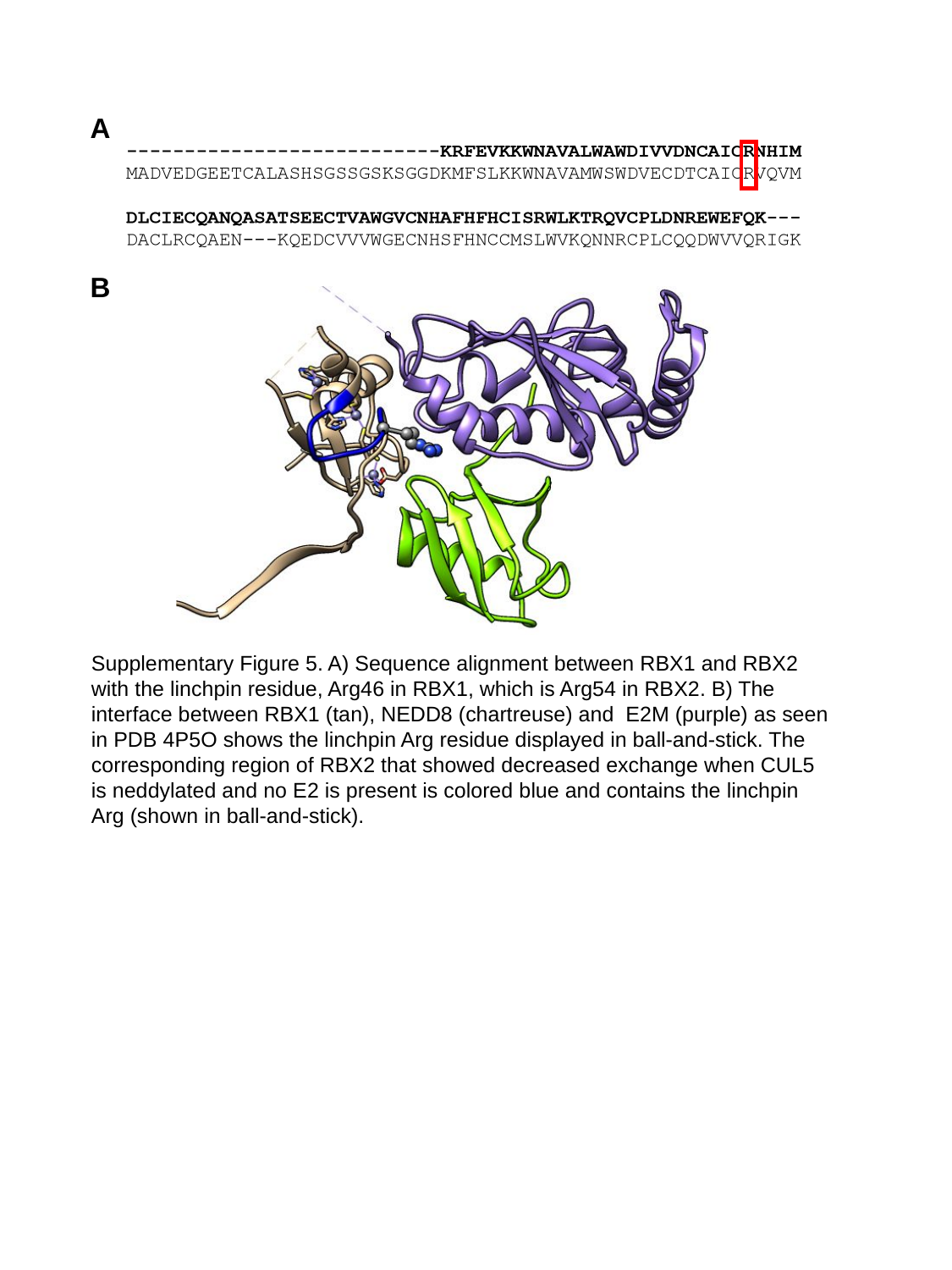

A
B
Supplementary Figure 5. A) Sequence alignment between RBX1 and RBX2 with the linchpin residue, Arg46 in RBX1, which is Arg54 in RBX2. B) The interface between RBX1 (tan), NEDD8 (chartreuse) and E2M (purple) as seen in PDB 4P5O shows the linchpin Arg residue displayed in ball-and-stick. The corresponding region of RBX2 that showed decreased exchange when CUL5 is neddylated and no E2 is present is colored blue and contains the linchpin Arg (shown in ball-and-stick).

## Slide 7
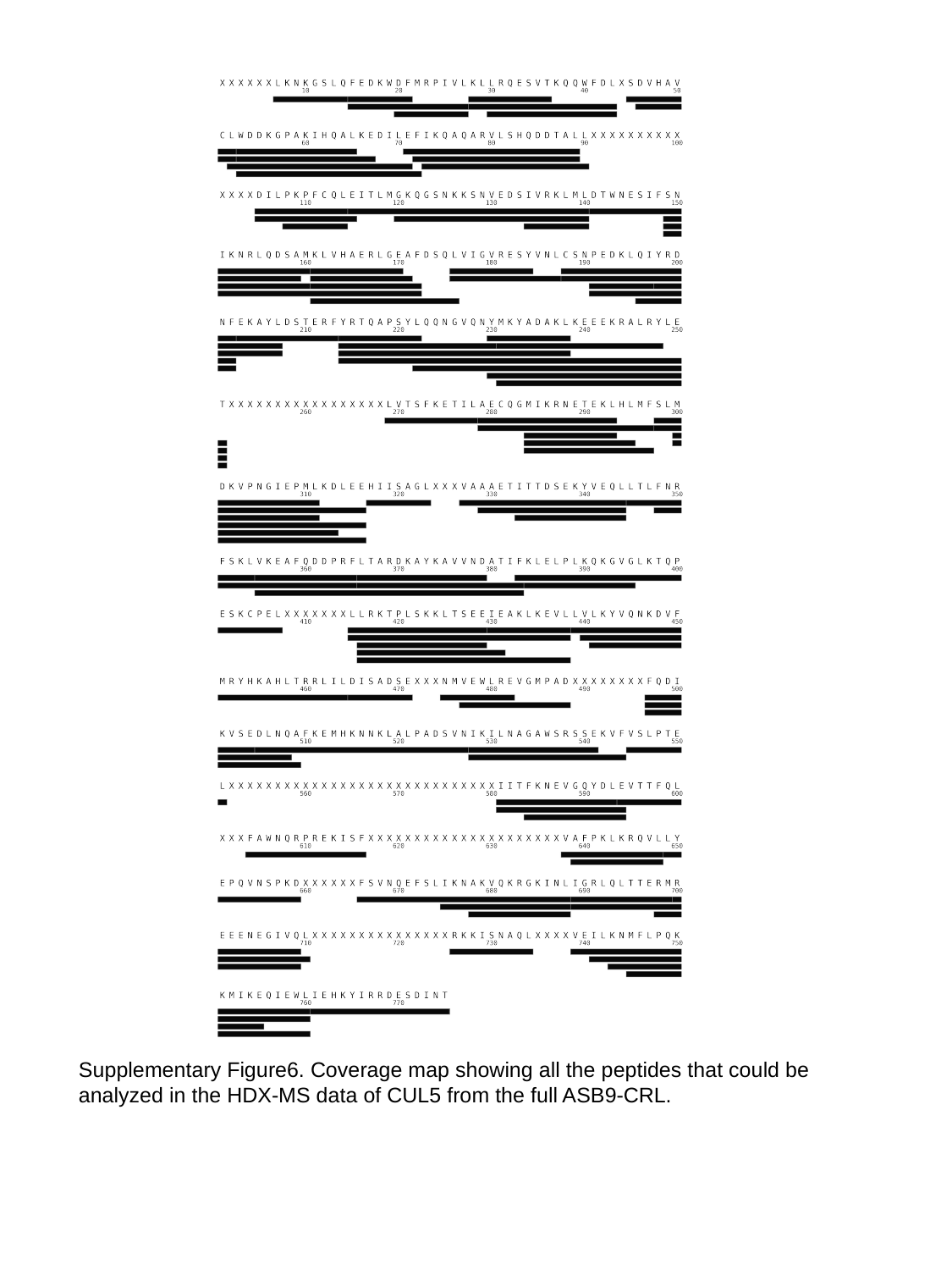

Supplementary Figure6. Coverage map showing all the peptides that could be analyzed in the HDX-MS data of CUL5 from the full ASB9-CRL.

## Slide 8
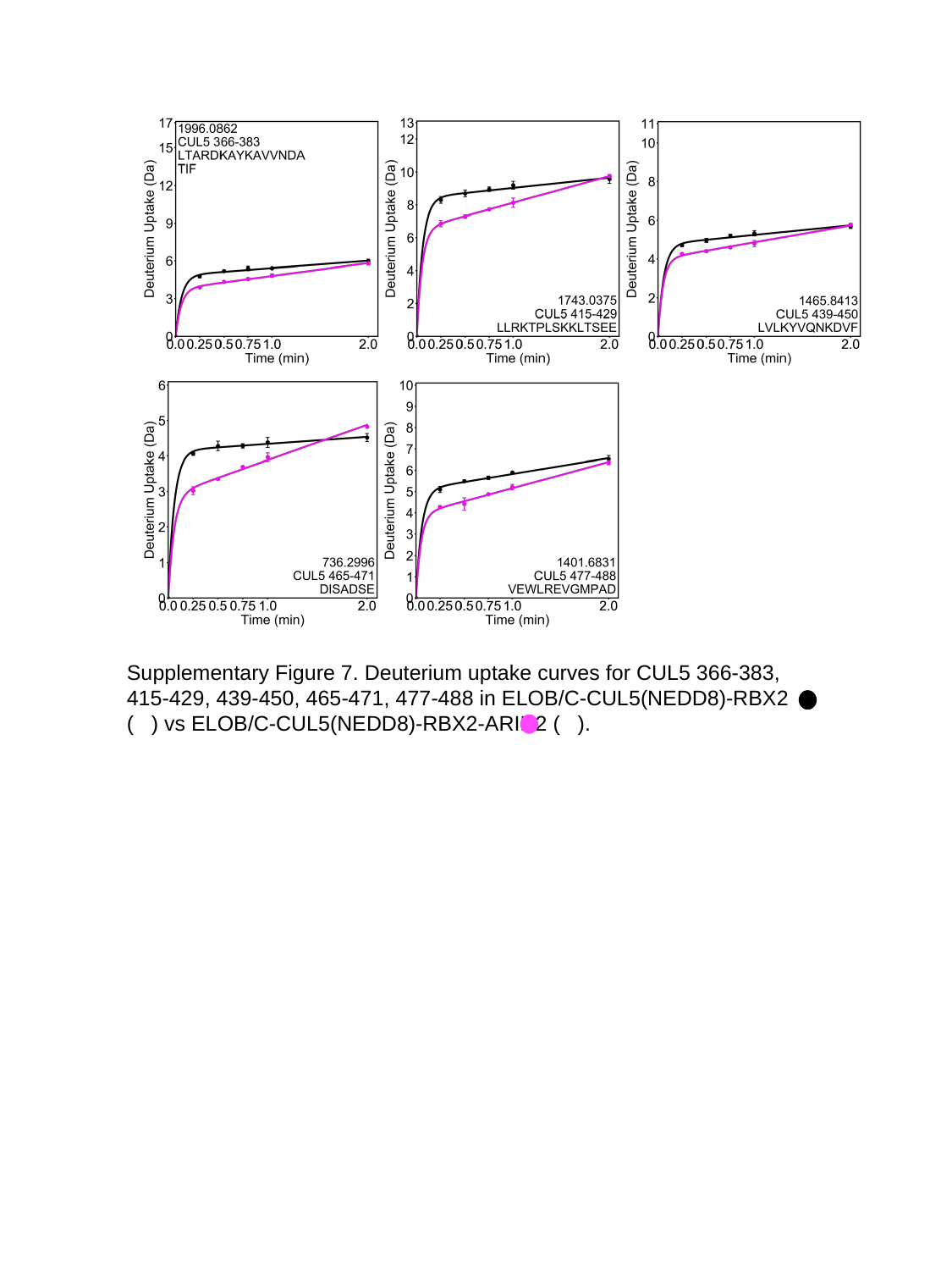

Supplementary Figure 7. Deuterium uptake curves for CUL5 366-383, 415-429, 439-450, 465-471, 477-488 in ELOB/C-CUL5(NEDD8)-RBX2 ( ) vs ELOB/C-CUL5(NEDD8)-RBX2-ARIH2 ( ).

## Slide 9
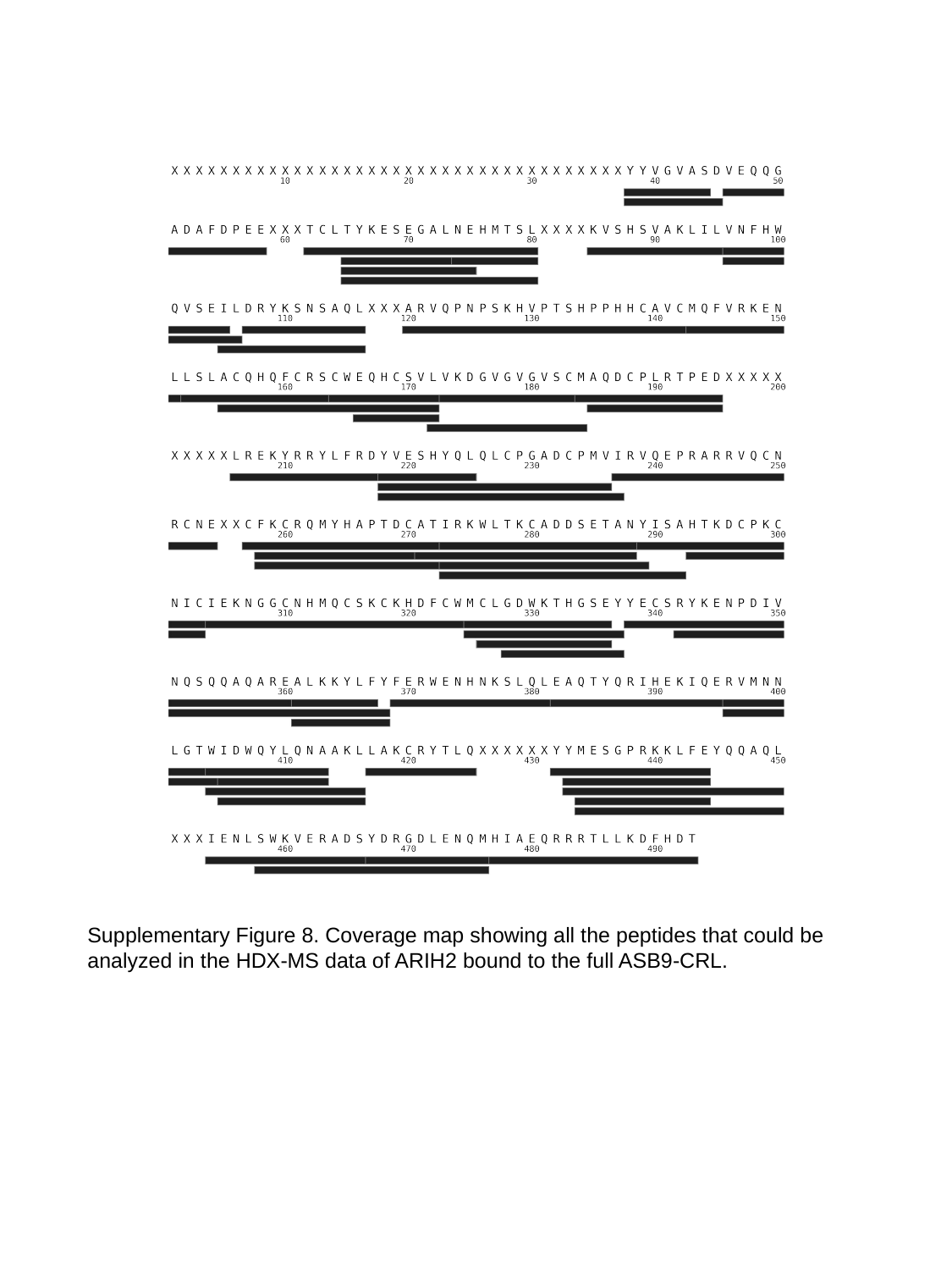

Supplementary Figure 8. Coverage map showing all the peptides that could be analyzed in the HDX-MS data of ARIH2 bound to the full ASB9-CRL.
